# Supplementary material for: Bioactive Compounds from an Endophytic Pezicula sp. Showing Antagonistic Effects against the Ash Dieback Pathogen
Source: Biomolecules. 2023 Nov 8;13(11):1632. doi: 10.3390/biom13111632 (PMC10669340; doi:10.3390/biom13111632)
Supplement: Supplementary file 1 [file biomolecules-13-01632-s001.zip › biomolecules-2648701-supplementary.pdf]

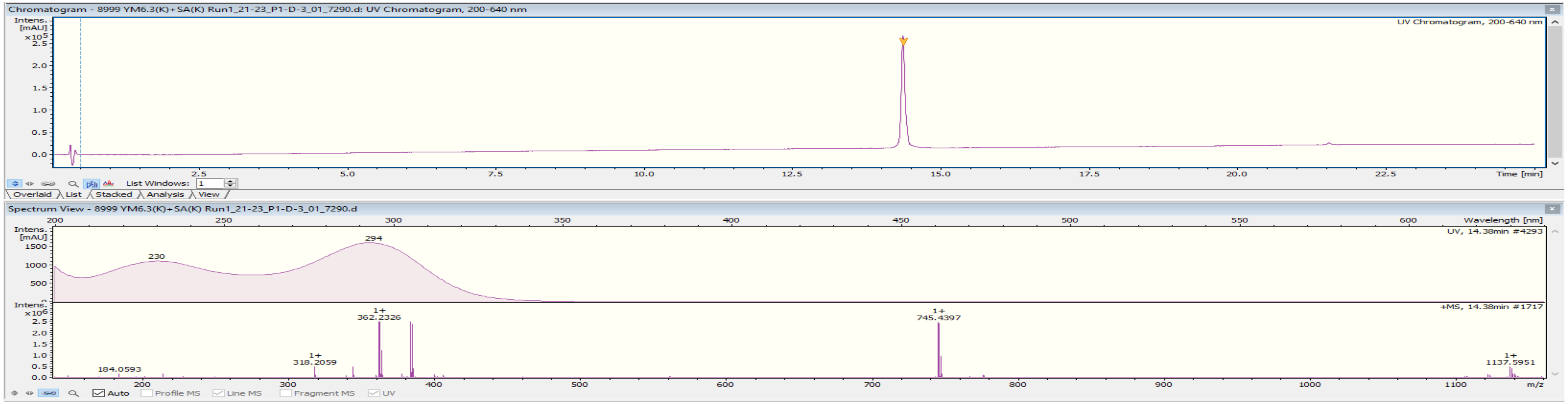

Figure S1: HR- ESI (+) MS data for CJ-17,572 (**1**)

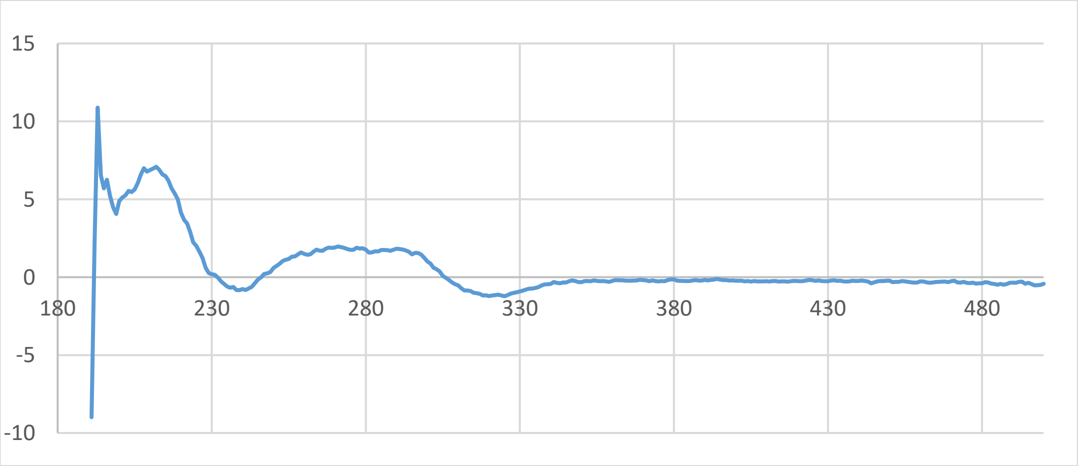

Figure S2: ECD spectrum (MeOH) of CJ-17,572 (**1**).

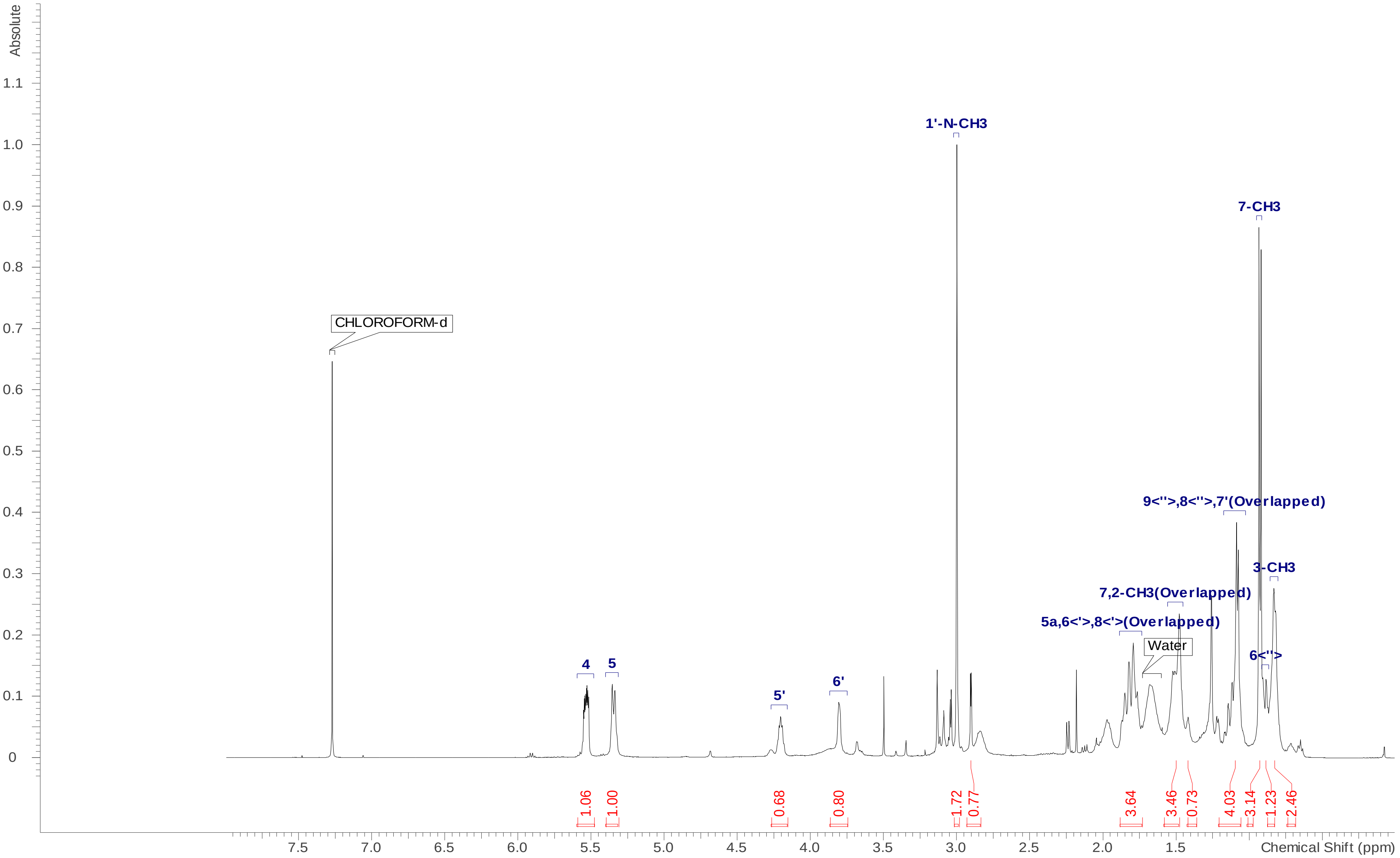

Figure S3: ^{1}H NMR spectrum (500 MHz, $CHCl_3$-*d*) of CJ-17,572 (**1**).

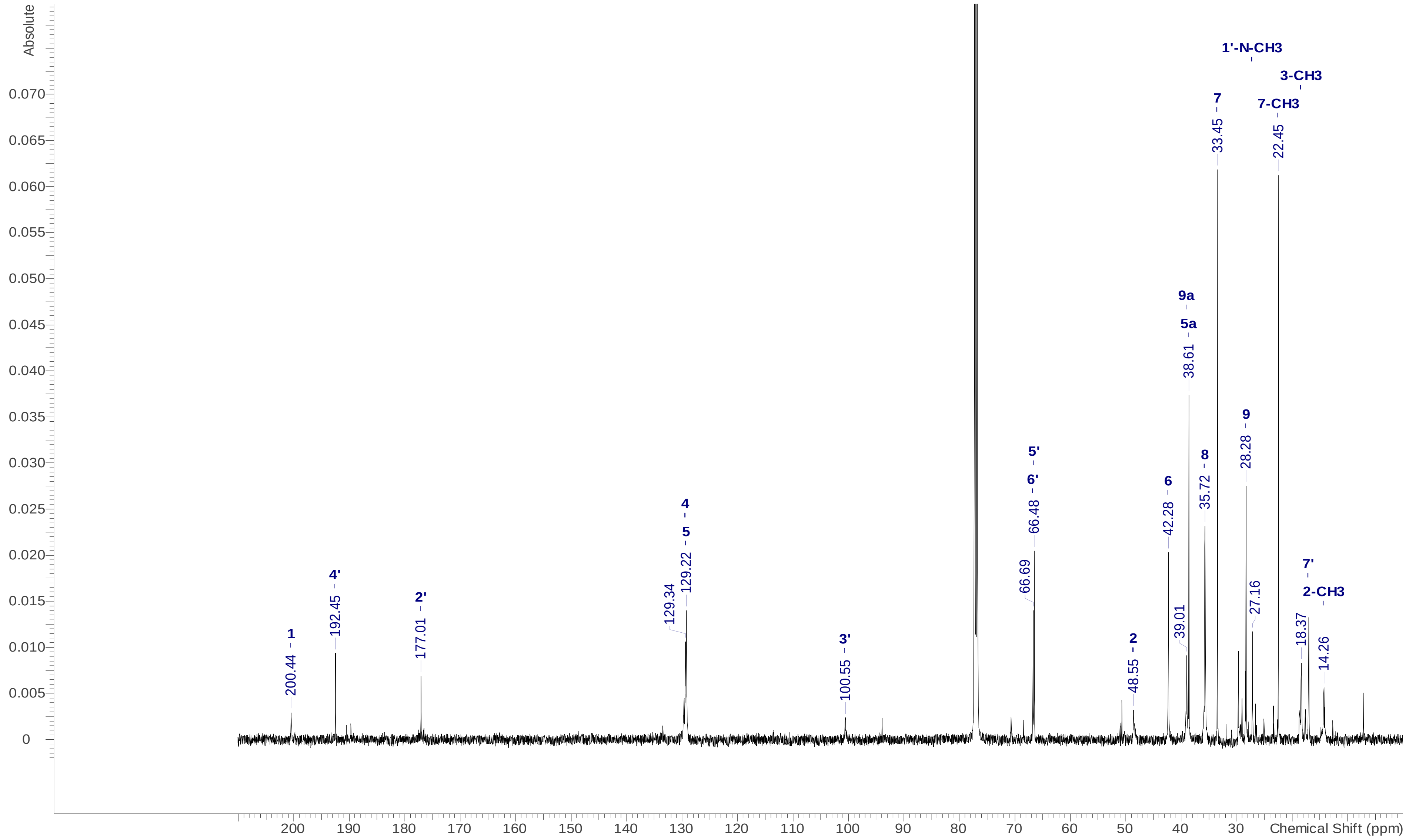

Figure S4: ^{13}C NMR spectrum (125 MHz, $CHCl_3$-*d*) of CJ-17,572 (**1**).

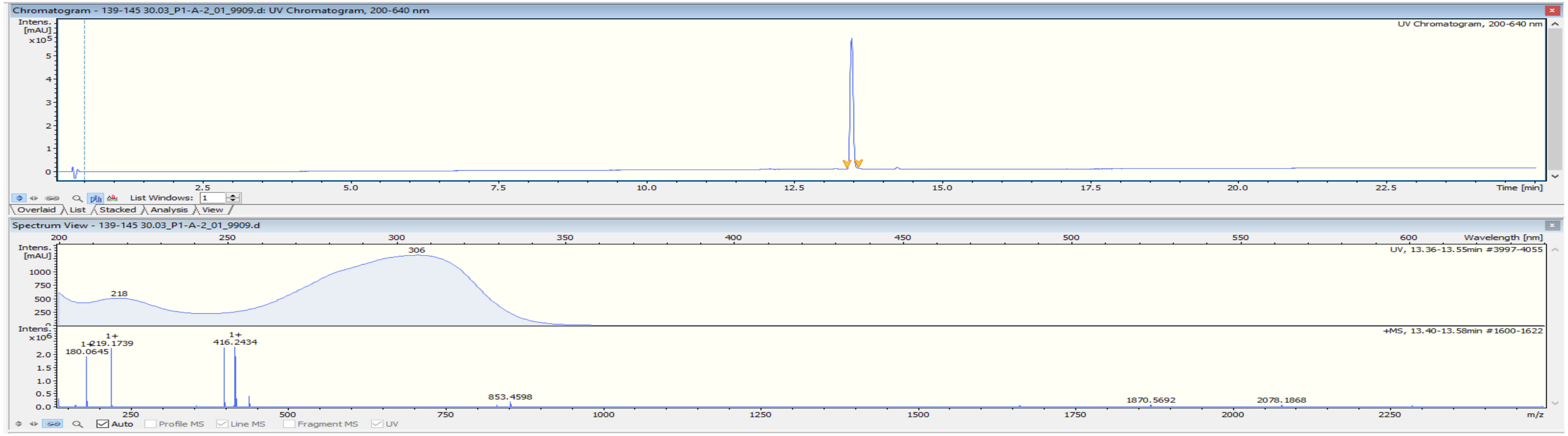

Figure S5: HR- ESI (+) MS data for peziculastatin (**2**).

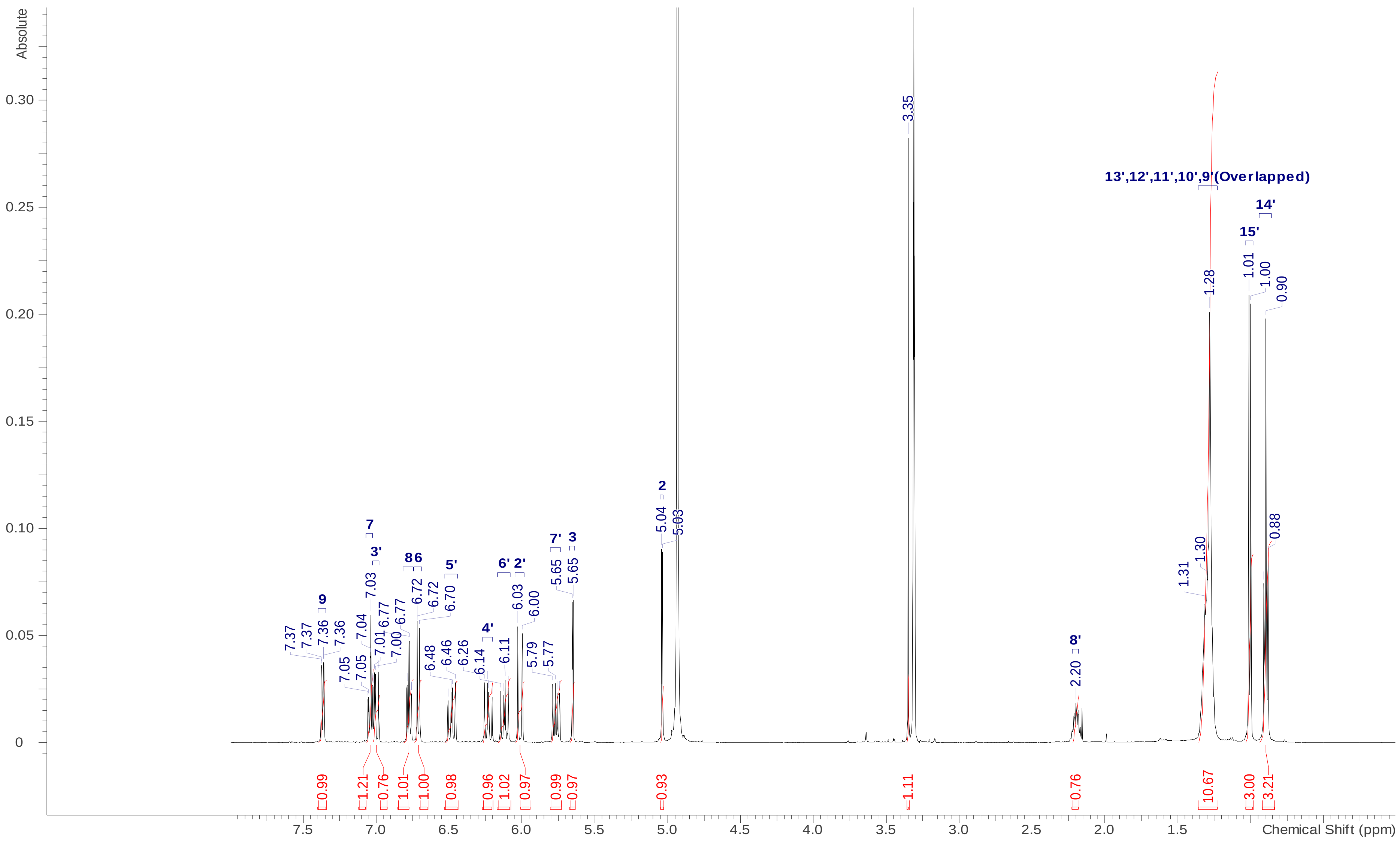

Figure S6: ^{1}H NMR spectrum (500 MHz, CH_3OH-d_4) of peziculastatin (**2**).

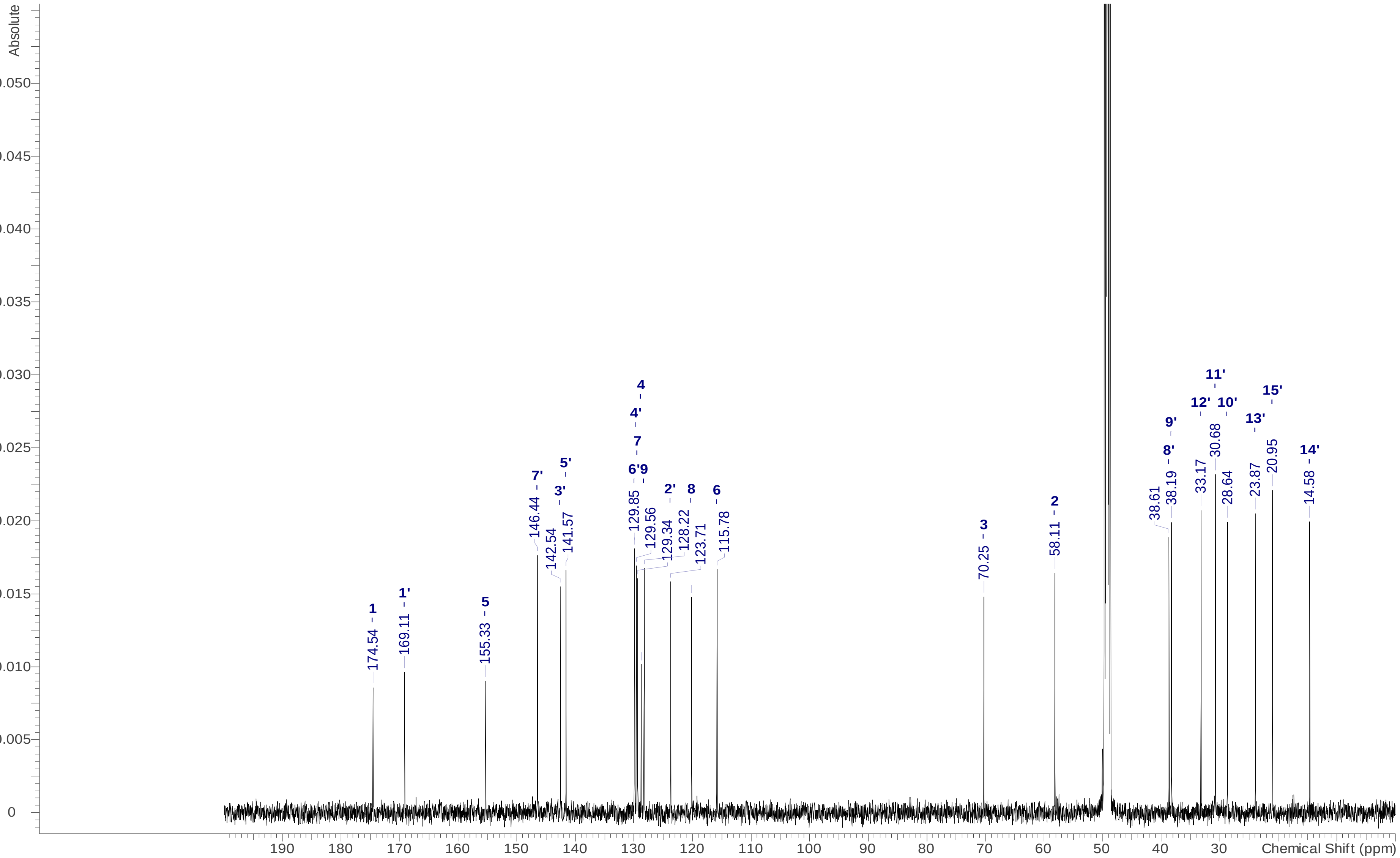

Figure S7: ^{13}C NMR spectrum (125 MHz, CH_3OH-*d*$_4$) of peziculastatin (**2**).

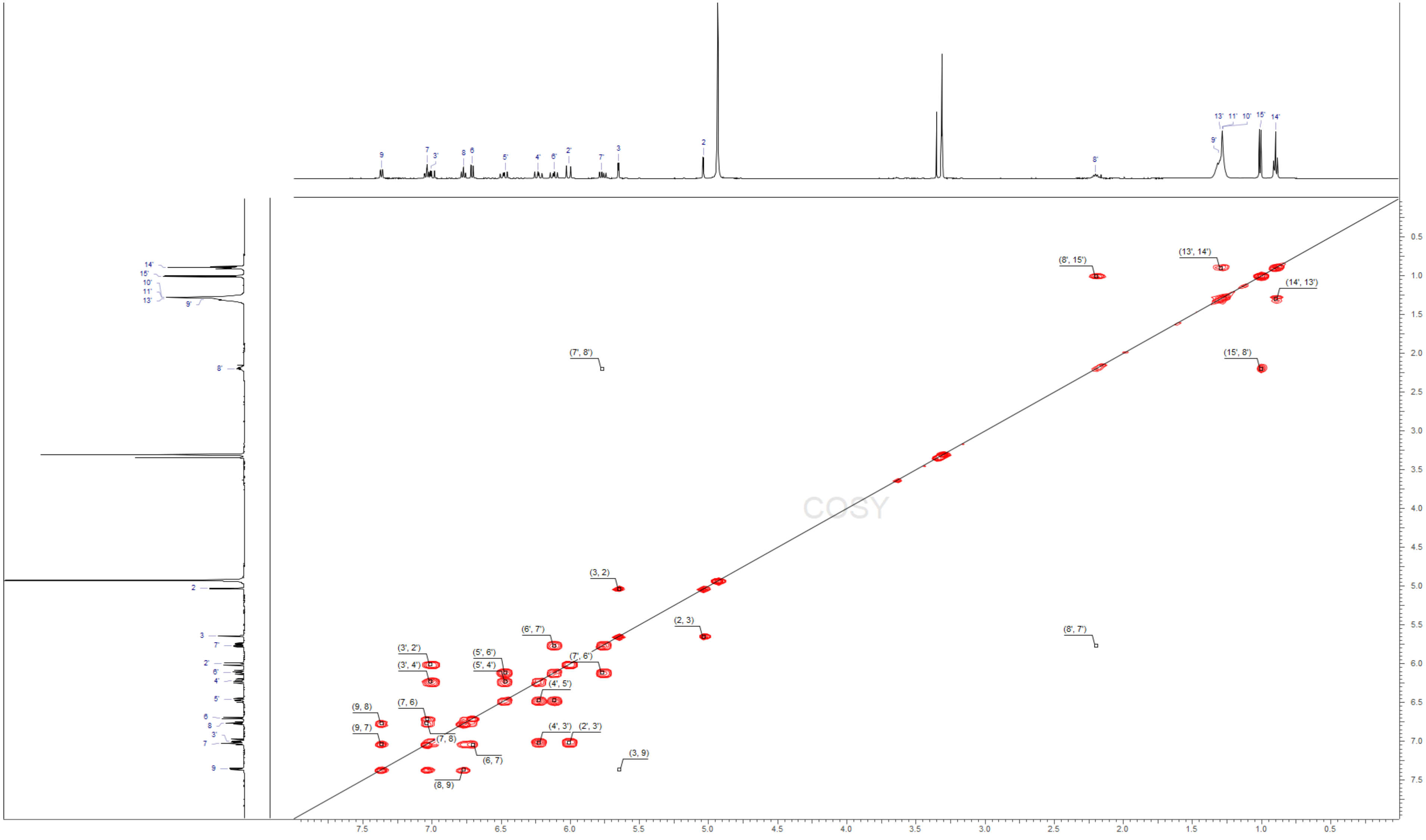

Figure S8: COSY NMR spectrum (500 MHz, CH_3OH-d_4) of peziculastatin (**2**).

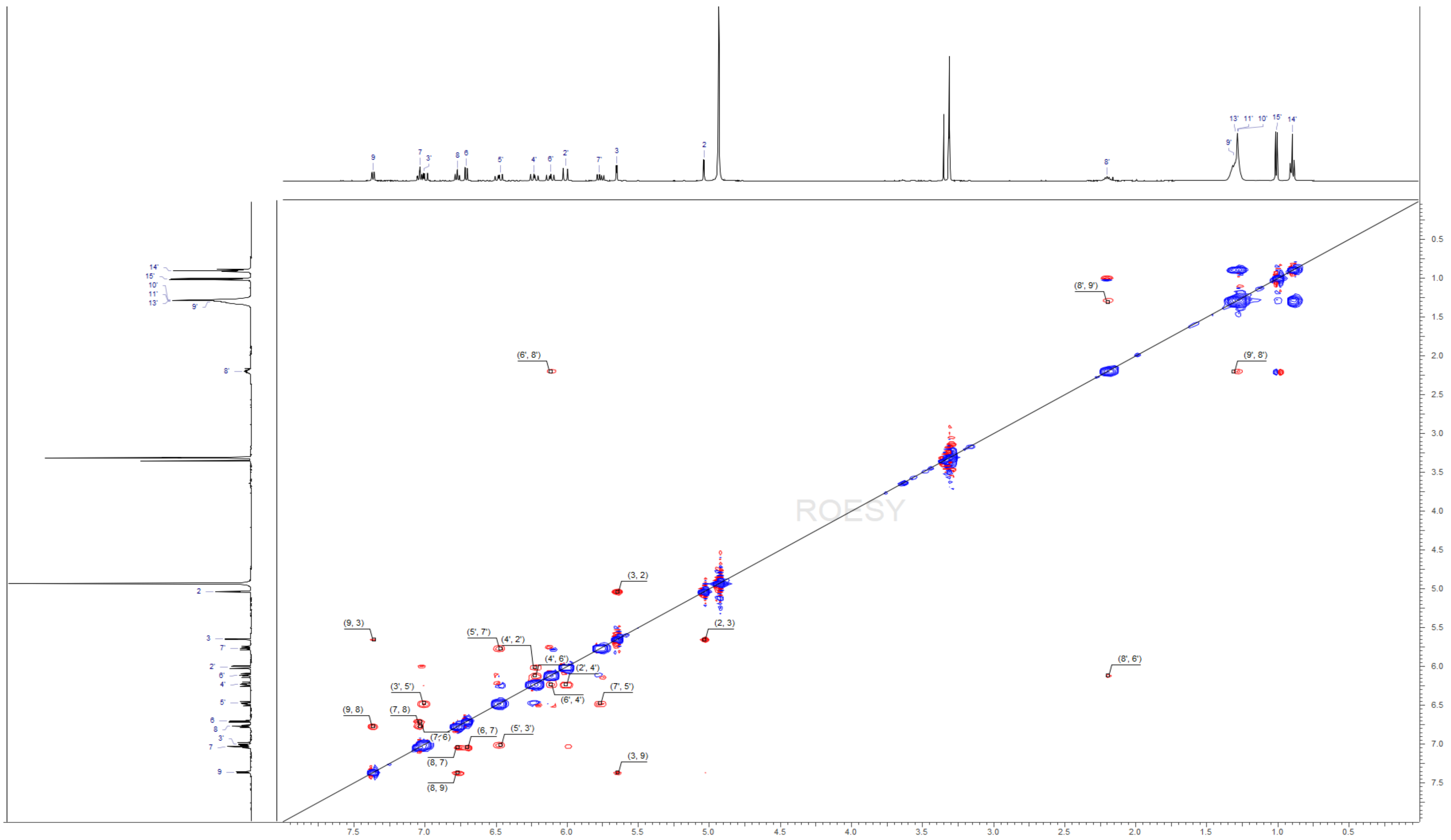

Figure S9: ROESY NMR spectrum (500 MHz, CH_3OH-d_4) of peziculastatin (**2**).

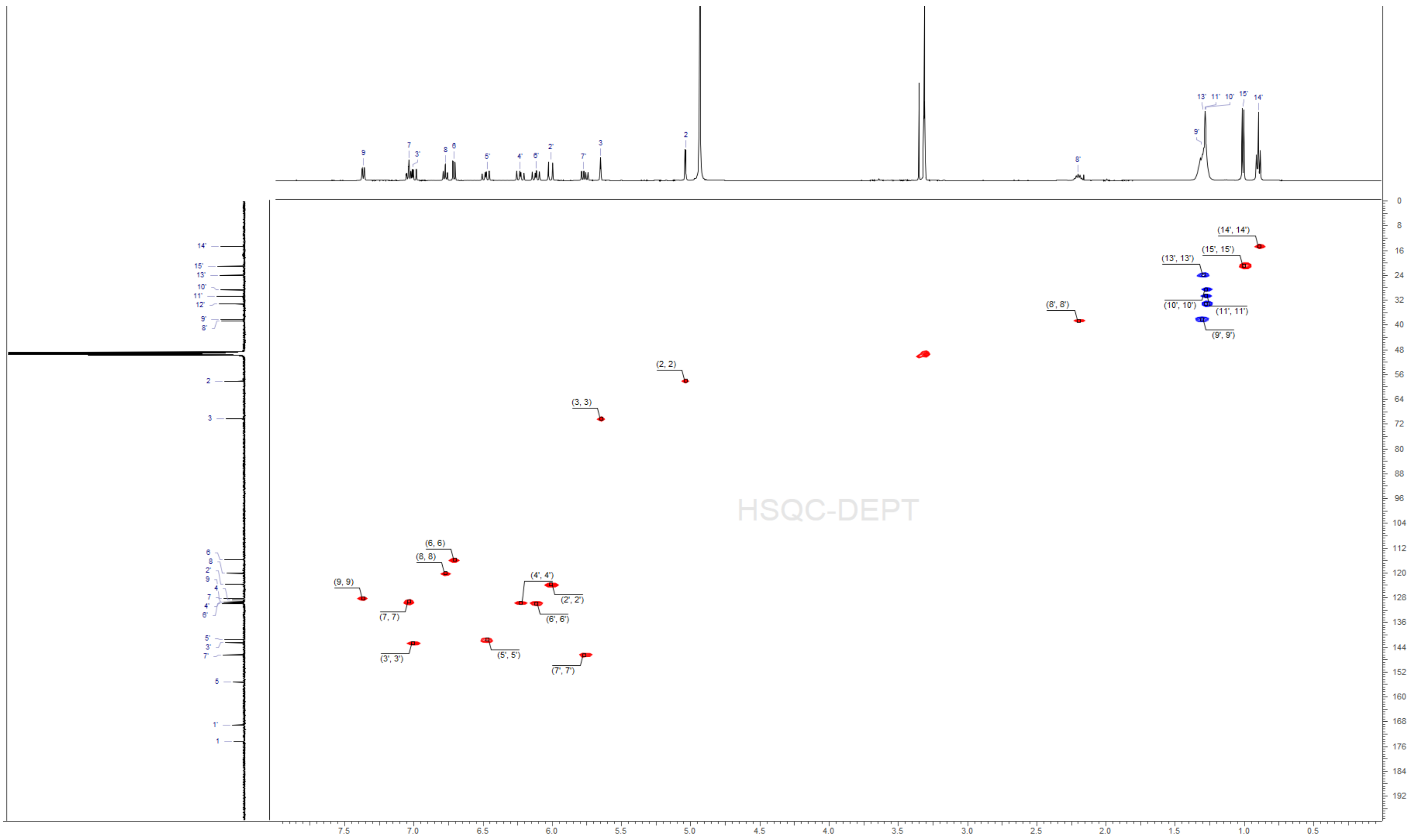

Figure S10: HSQC NMR spectrum (500 MHz, CH_3OH-d_4) of peziculastatin (**2**).

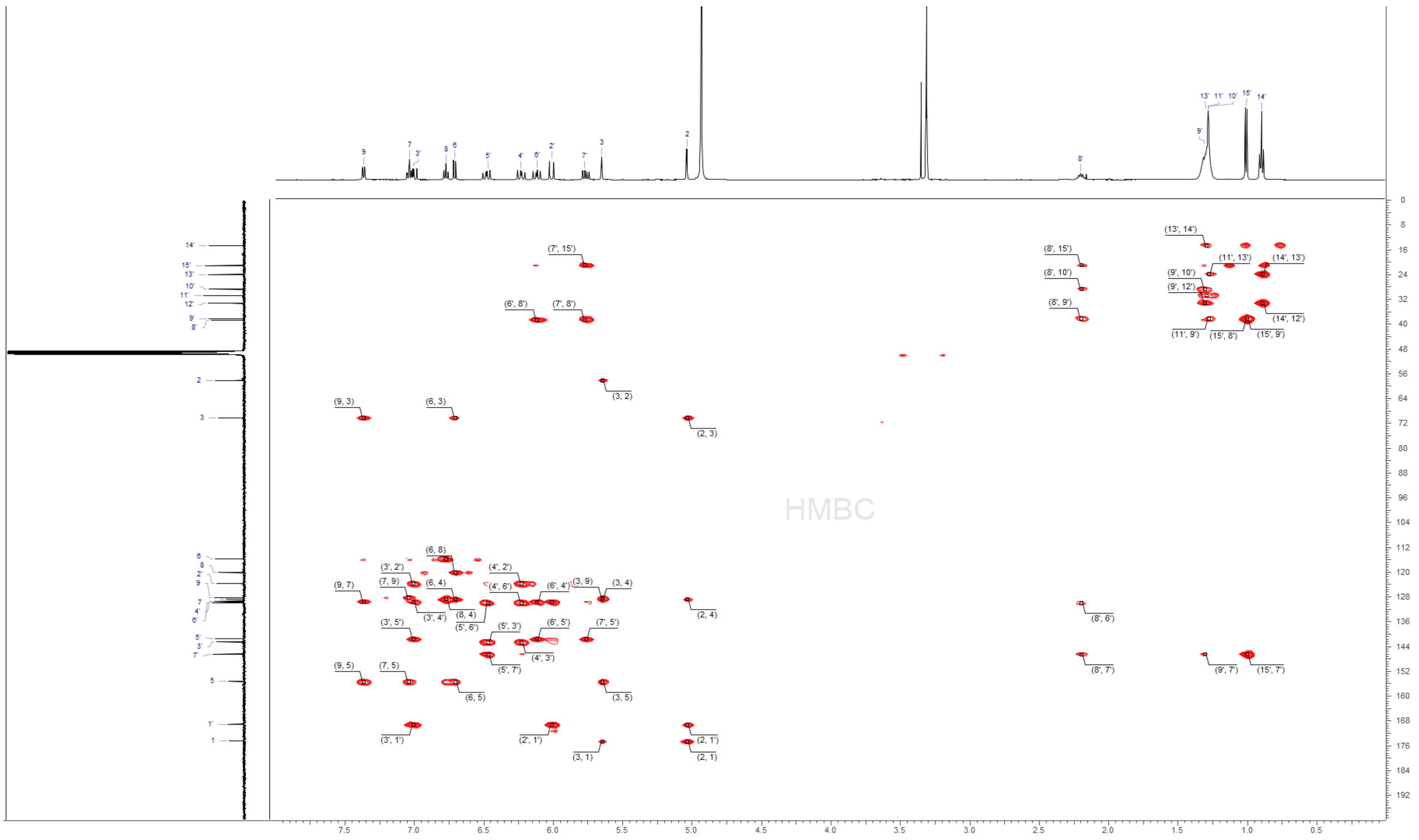

Figure S11: HMBC NMR spectrum (500 MHz, CH_3OH-d_4) of peziculastatin (**2**).

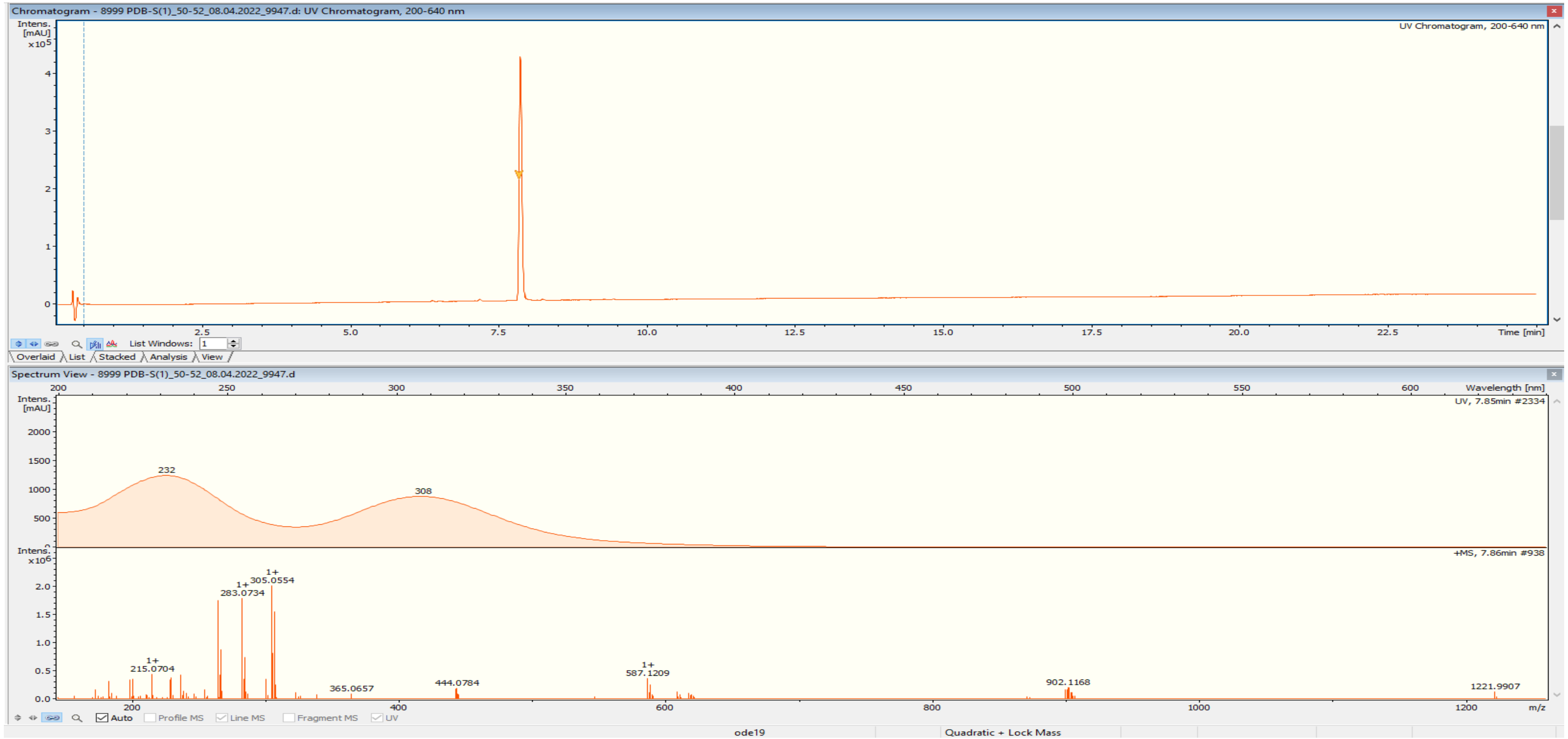

Figure S12: HR- ESI (+) MS data for mycorrhizin A (**3**).

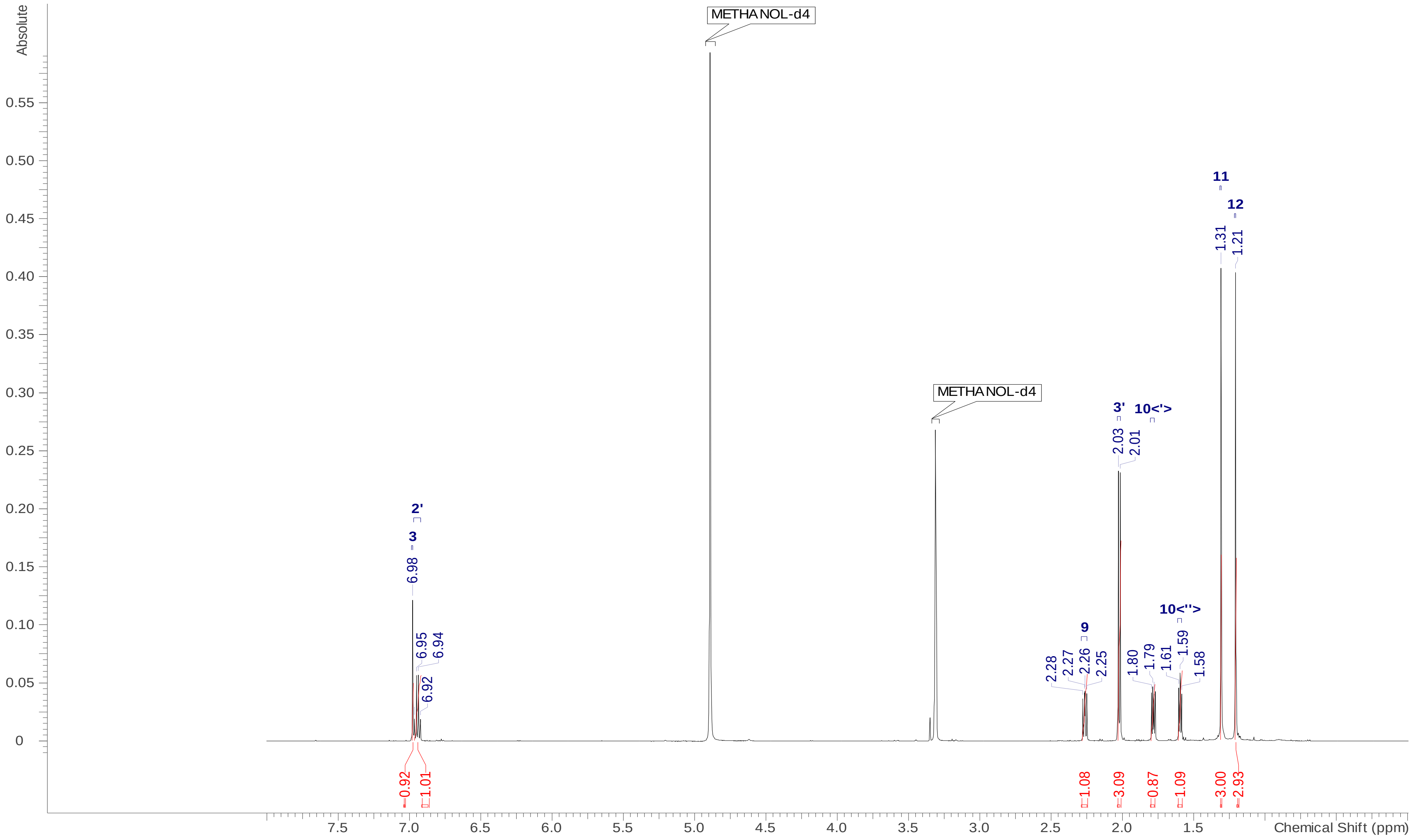

Figure S13: ^{1}H NMR spectrum (500 MHz, CH_3OH-d_4) of mycorrhizin A (**3**).

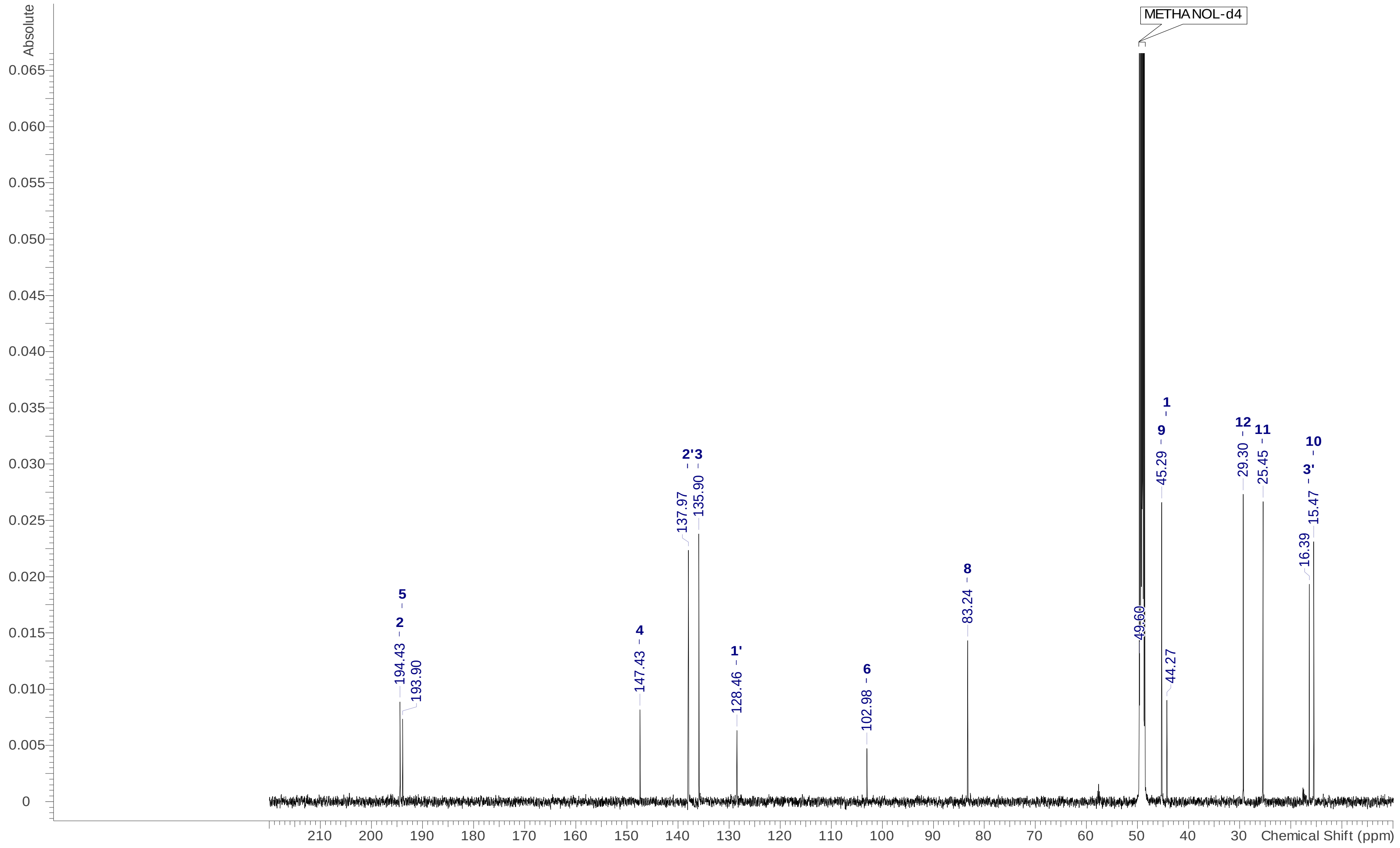

Figure S14: ^{13}C NMR spectrum (125 MHz, CH_3OH-d_4) of mycorrhizin A (**3**).

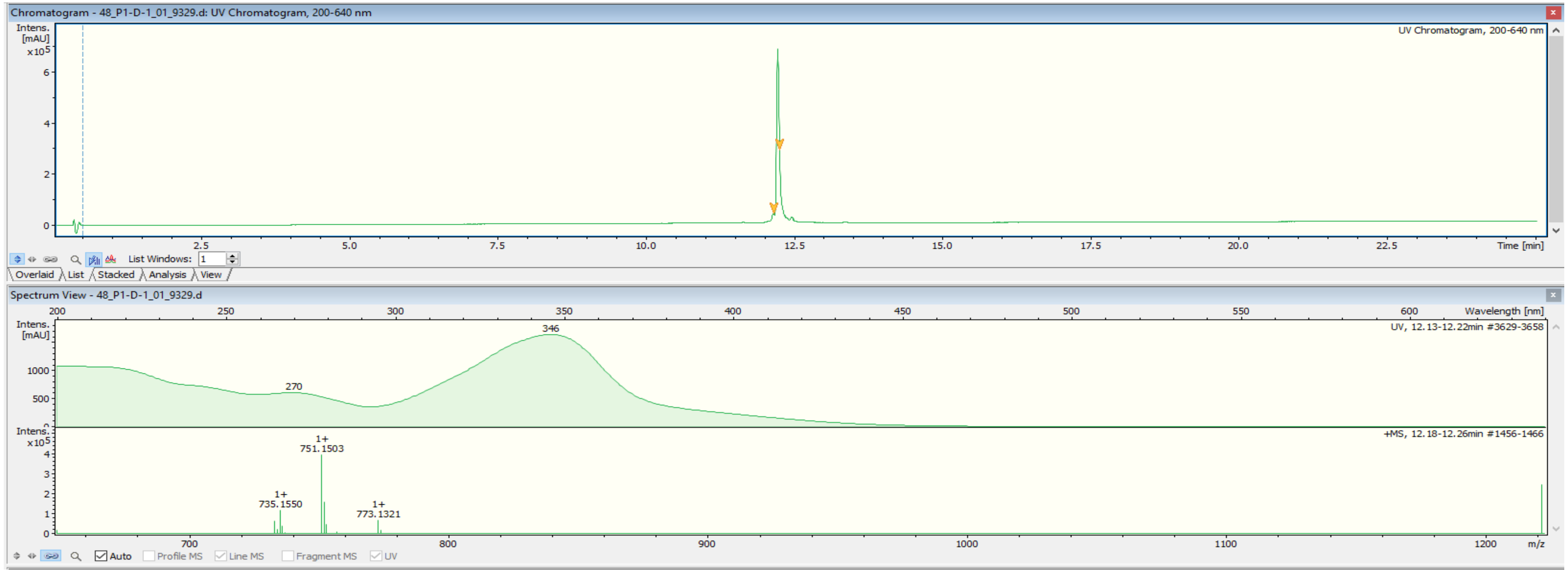

Figure S15: HR- ESI (+) MS data for cryptosporioptide A (**4**).

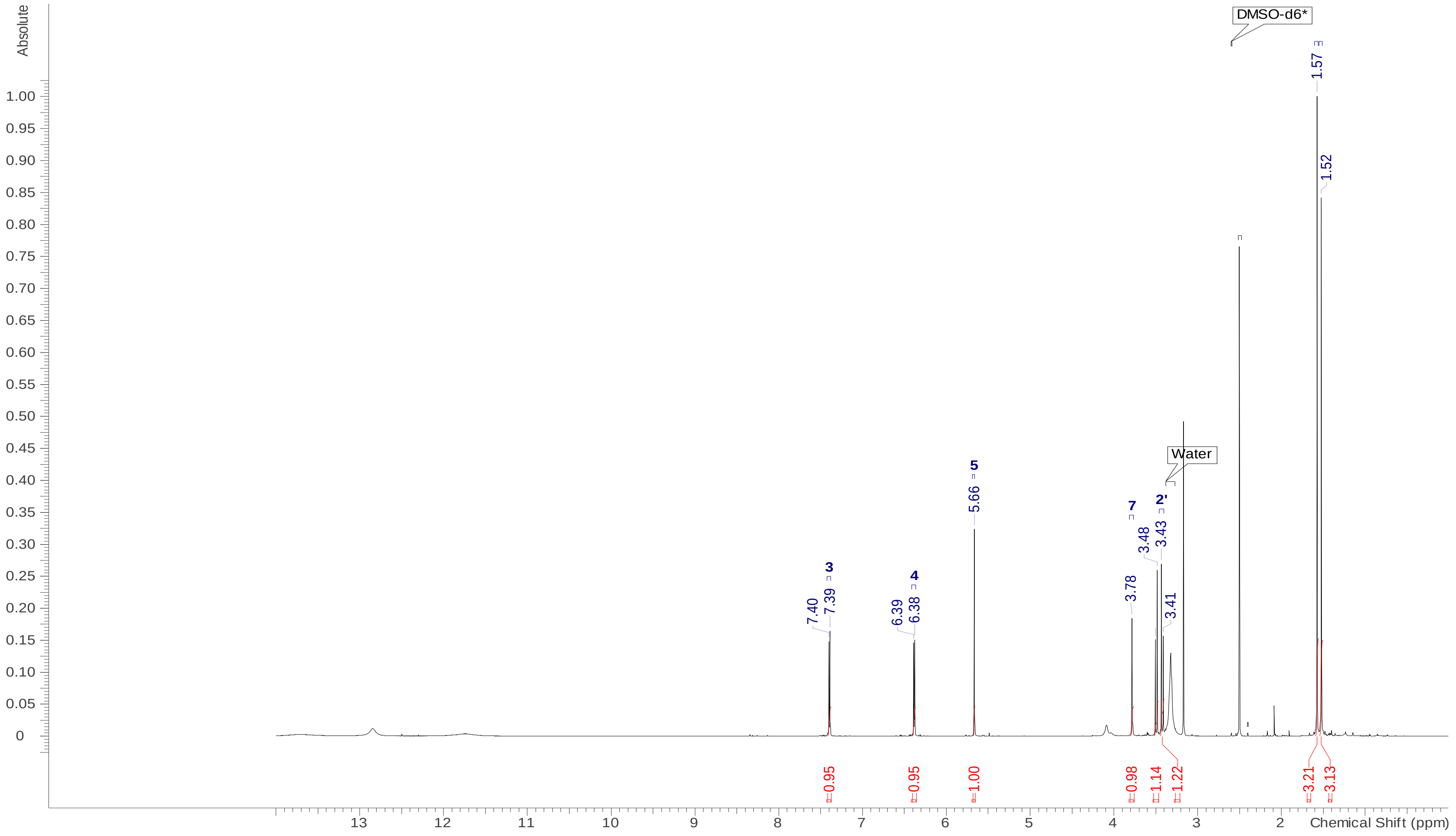

Figure S16: ^{1}H NMR spectrum (700 MHz, DMSO-*d*$_6$) of cryptosporioptide A (**4**).

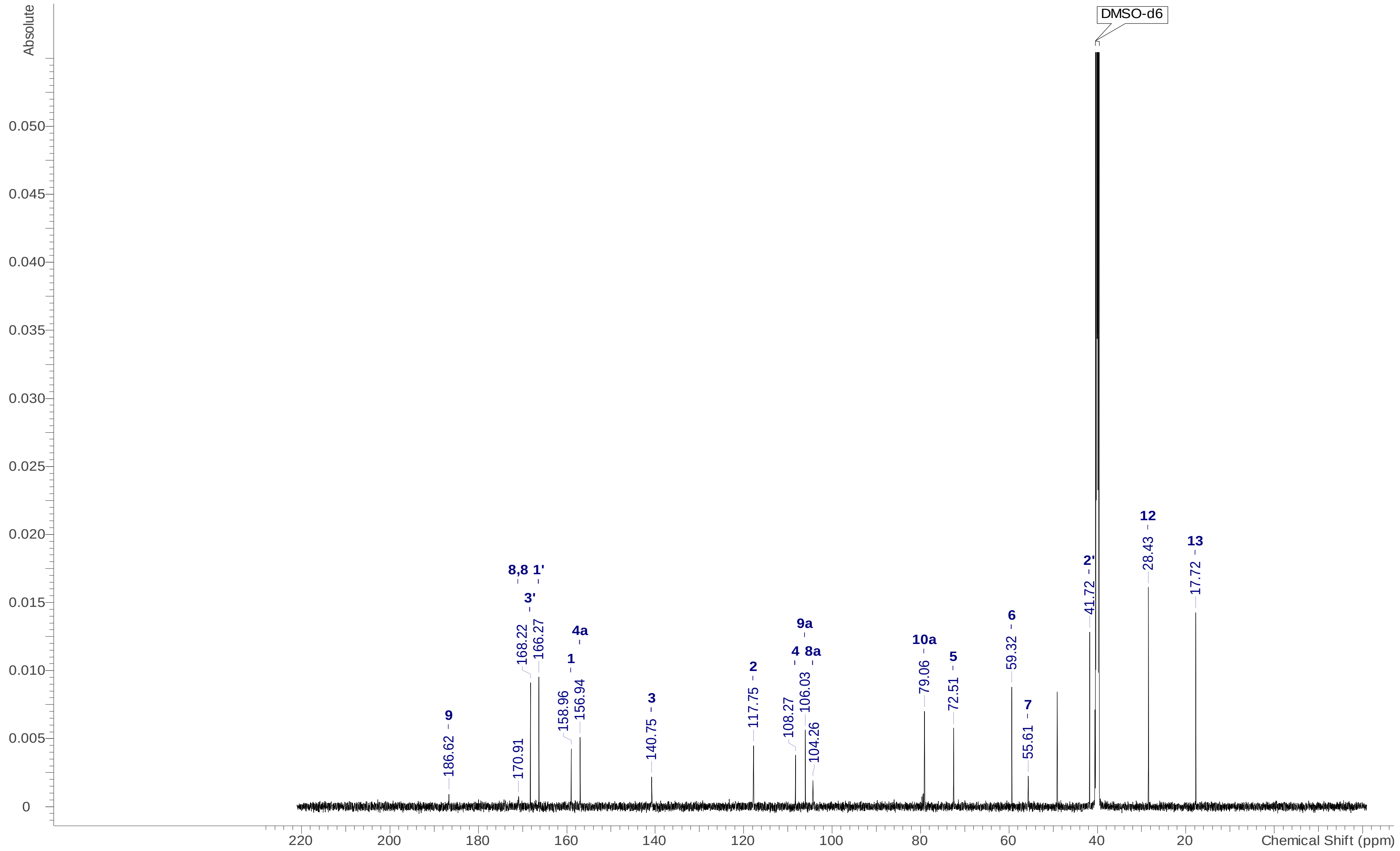

Figure S17: ^{1}H NMR spectrum (175 MHz, DMSO-*d*6) of cryptosporioptide A (**4**).

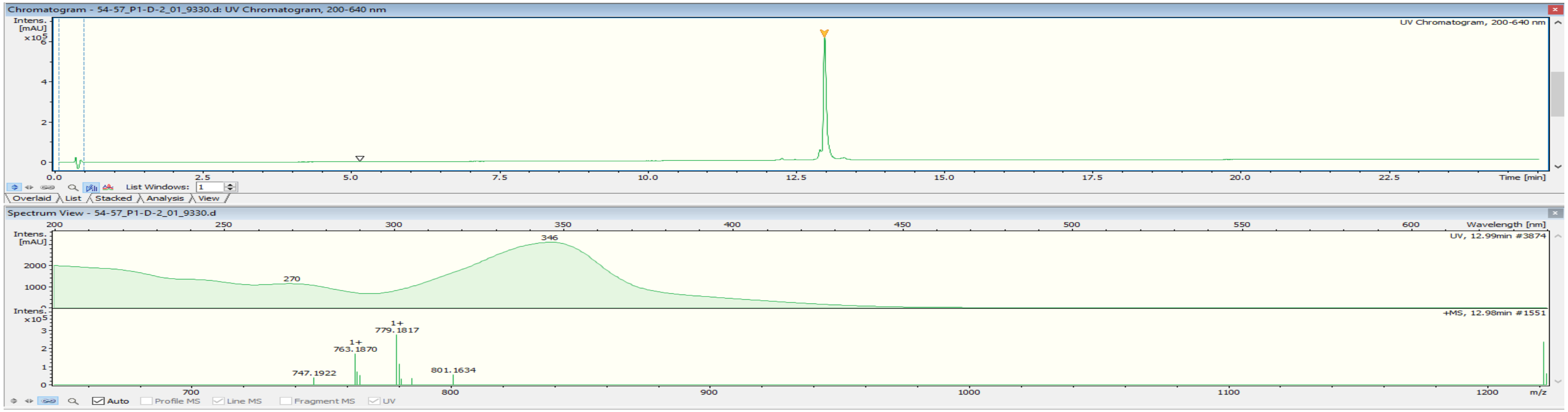

Figure 18: HR- ESI (+) MS data for cryptosporioptide B (**5**).

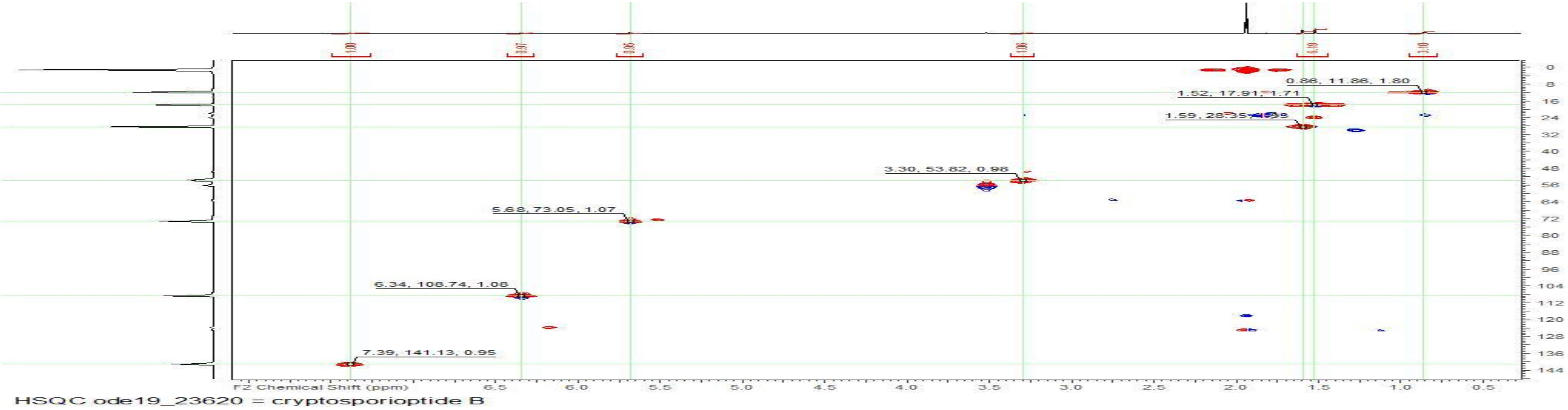

Figure S19: HSQC NMR spectrum (700 MHz, acetone-d_6) of cryptosporioptide B (**5**).

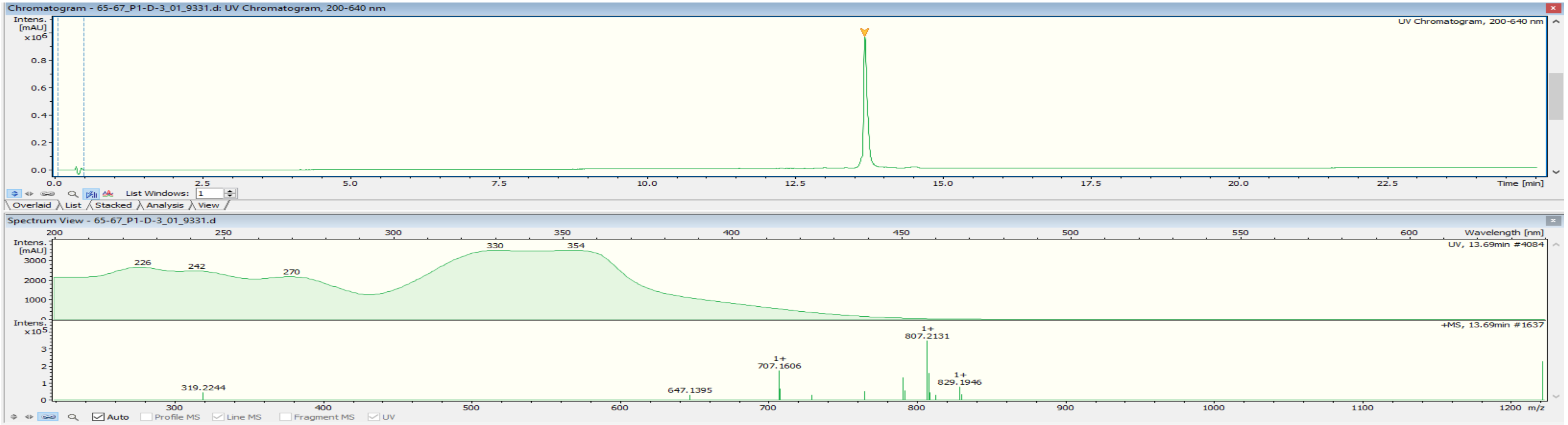

Figure S20: HR- ESI (+) MS data for cryptosporioptide C (**6**).

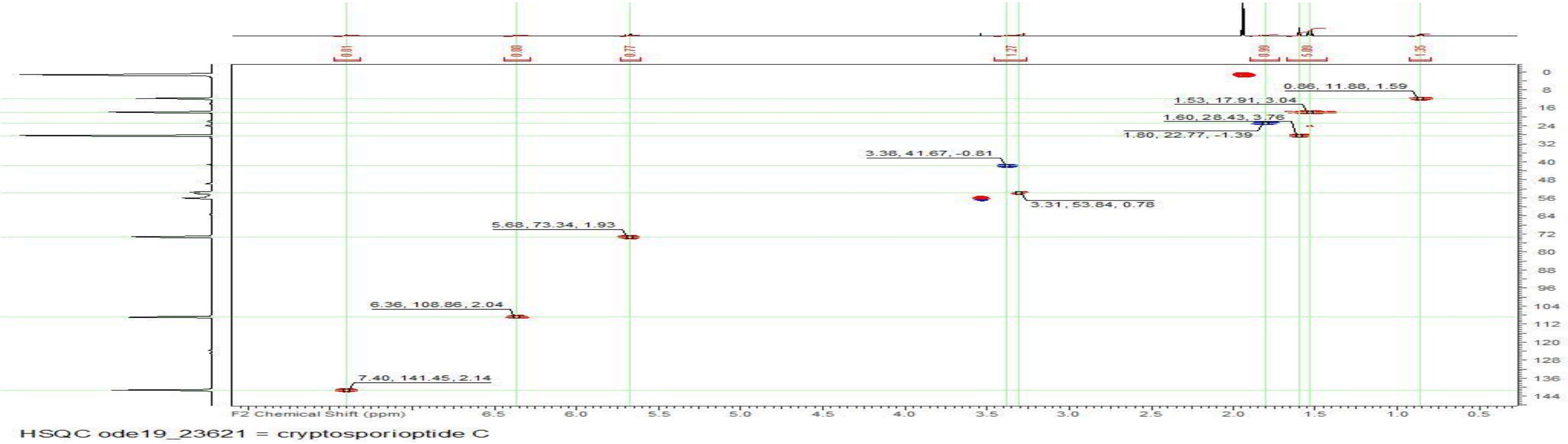

Figure S21: HSQC NMR spectrum (700 MHz, acetone-*d*6) of cryptosporioptide C (**6**).

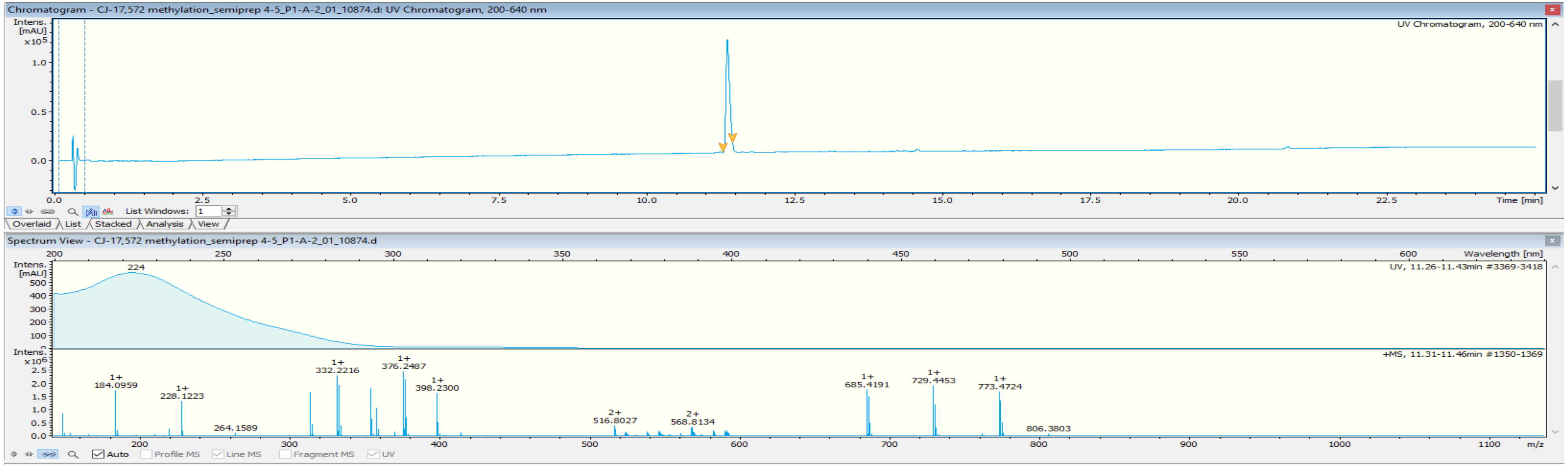

Figure S22: HR- ESI (+) MS data for (**7**).

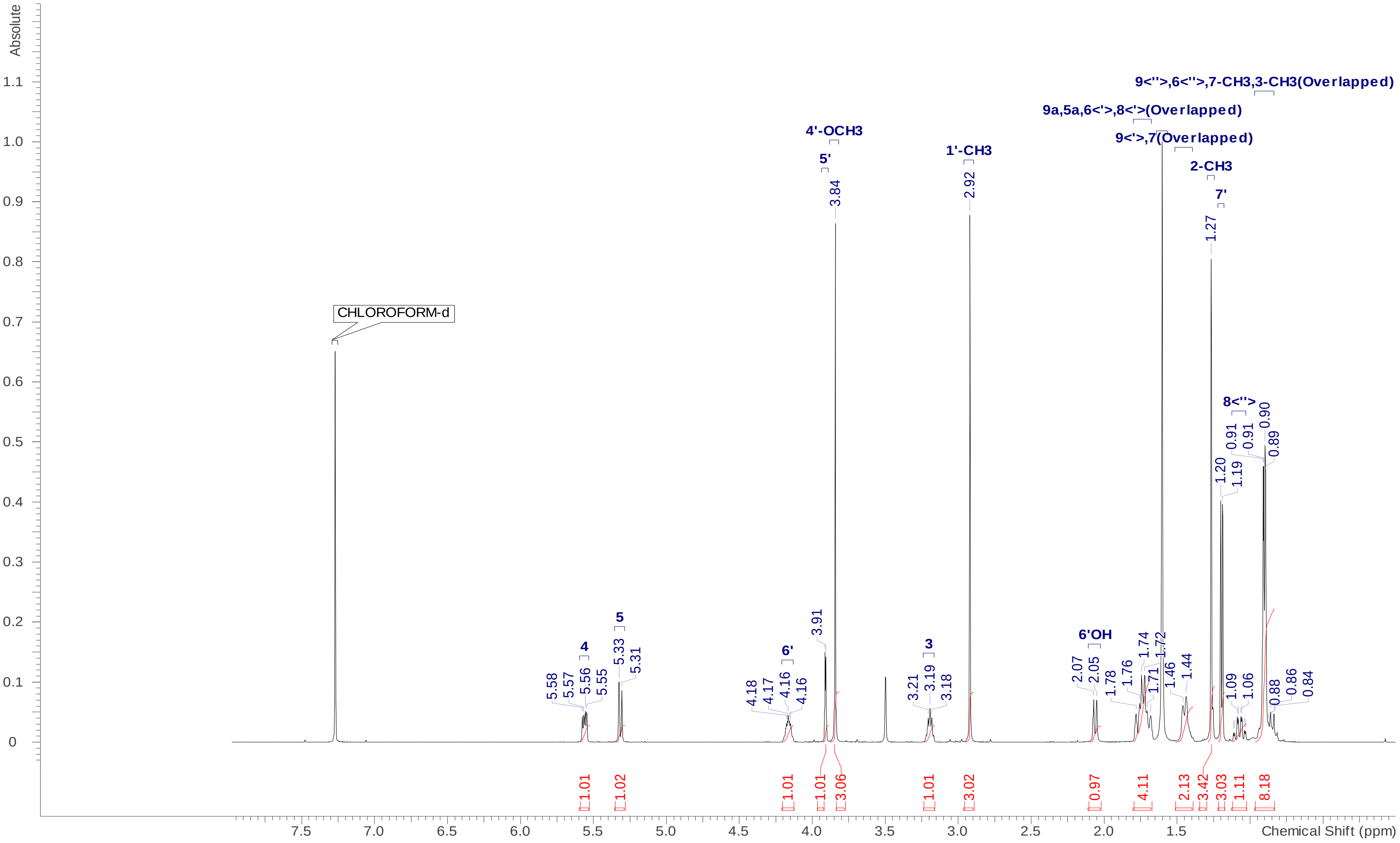

Figure S23: ^{1}H NMR spectrum (500 MHz, $CHCl_3$-*d*) of 4′-methyl-CJ-17,572 (**7**).

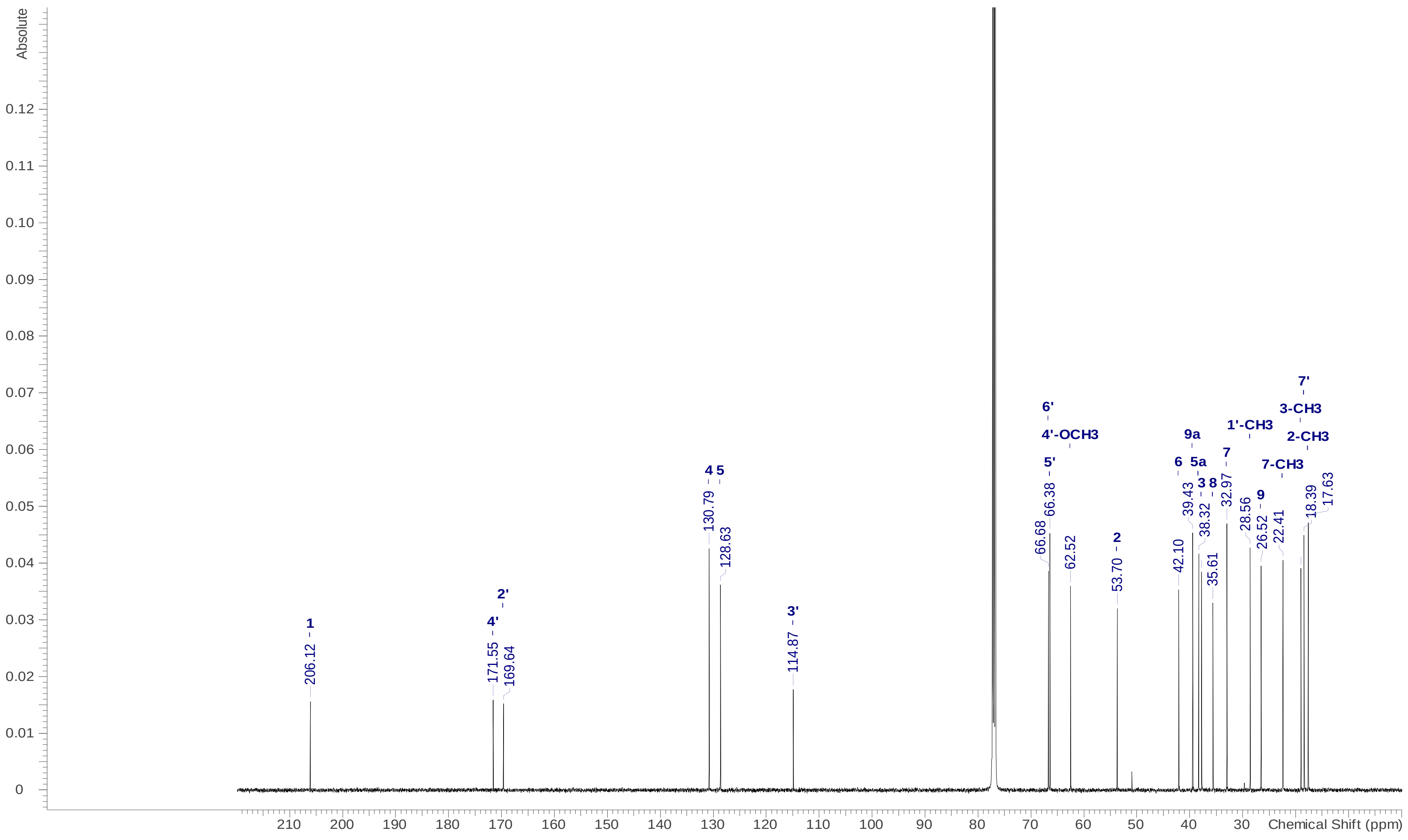

Figure S24: ^{13}C NMR spectrum (125 MHz, $CHCl_3$-*d*) of 4′-methyl-CJ-17,572 (**7**).

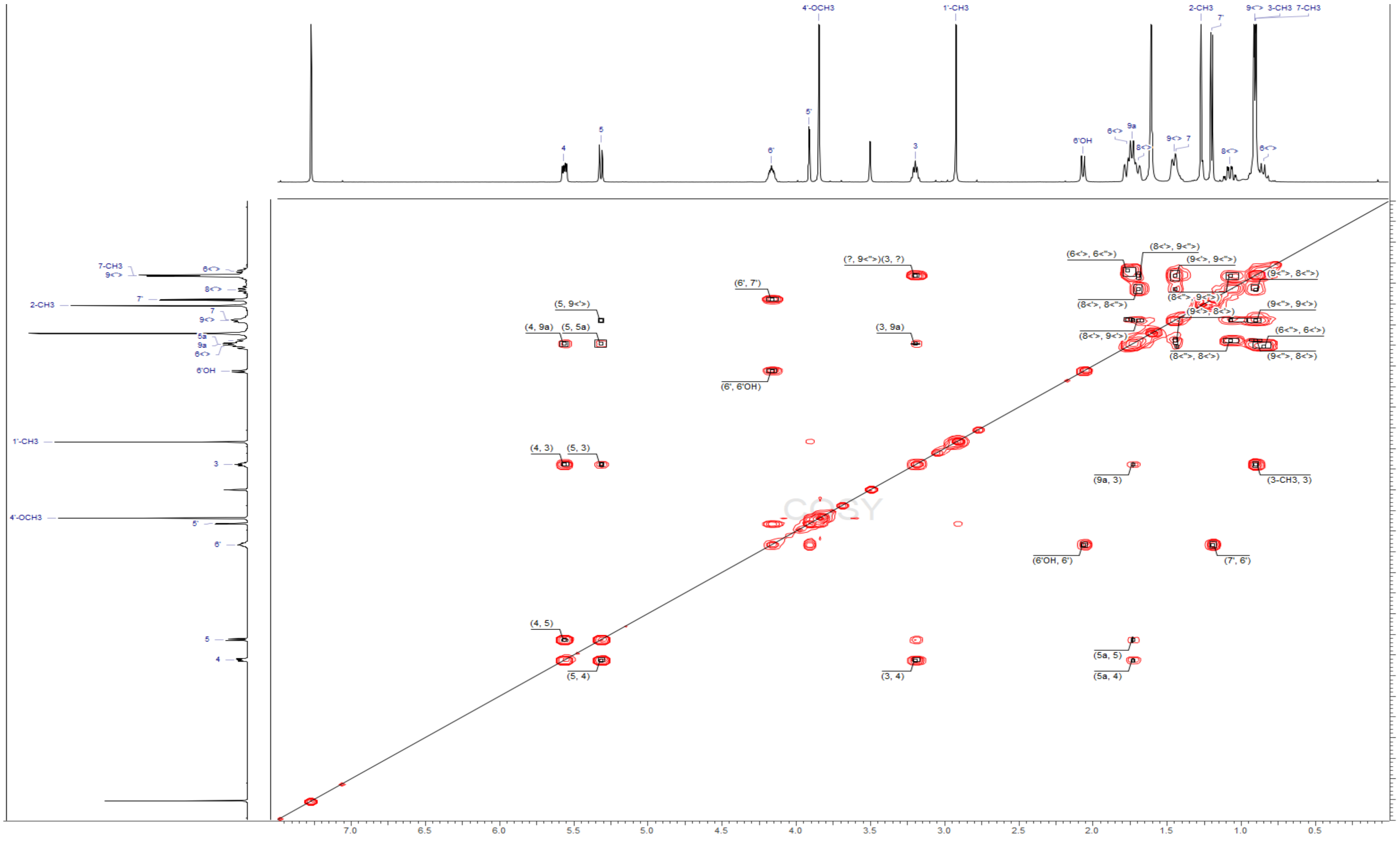

Figure S25: COSY NMR spectrum (500 MHz, $CHCl_3$-*d*) of 4′-methyl-CJ-17,572 (**7**).

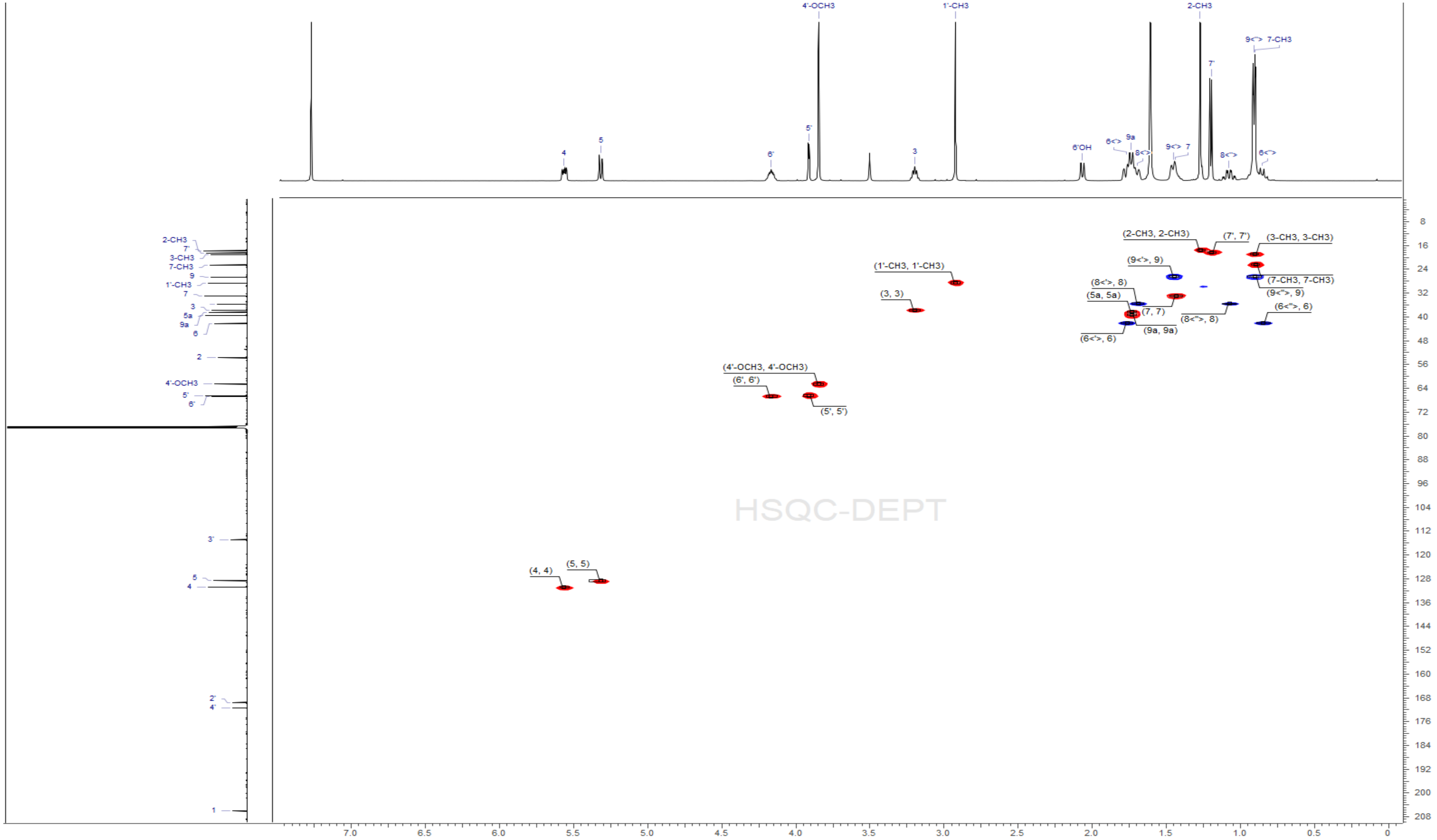

Figure S26: COSY NMR spectrum (500 MHz, $CHCl_3$-*d*) of 4′-methyl-CJ-17,572 (**7**).

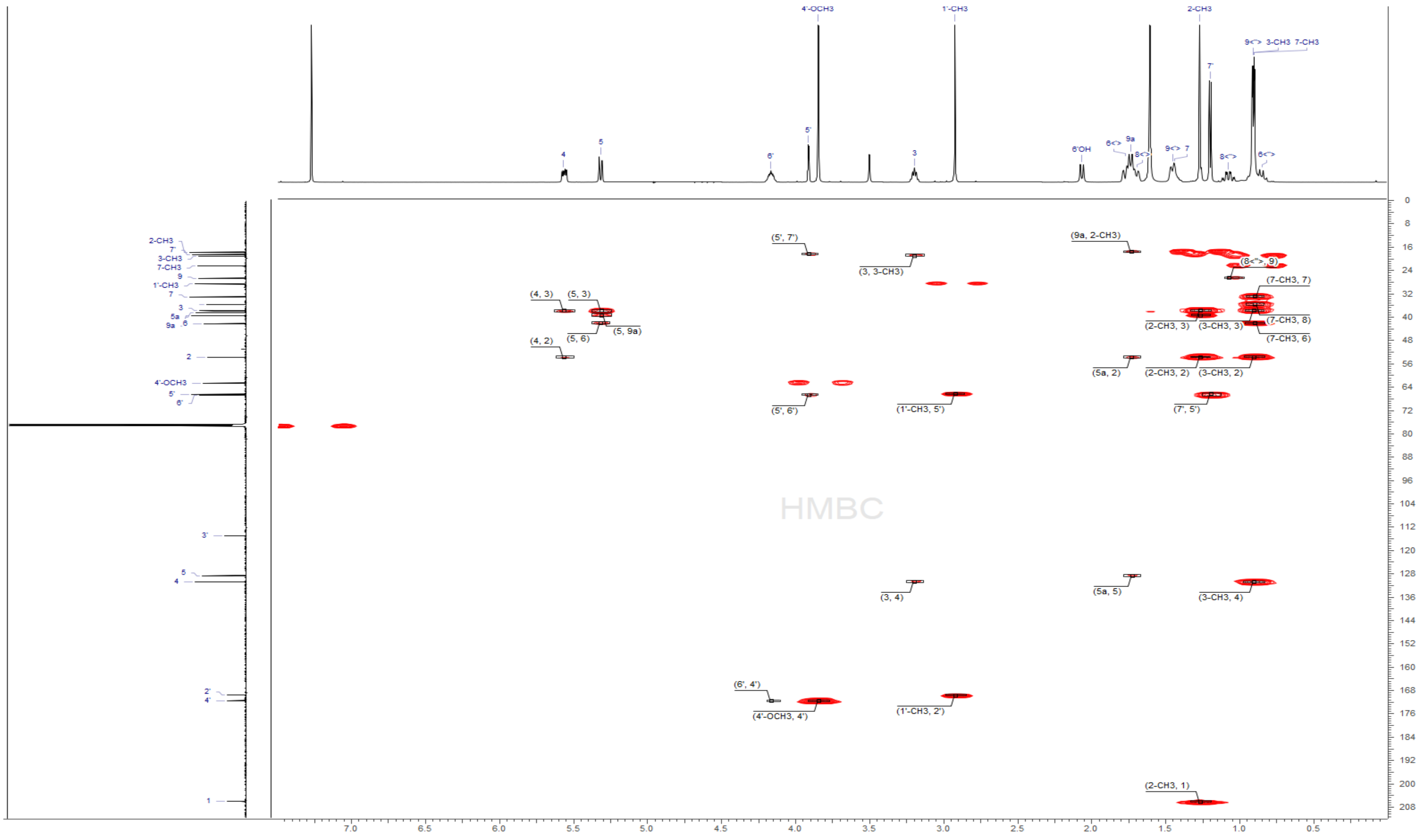

Figure S27: HMBC NMR spectrum (500 MHz, $CHCl_3$-*d*) of 4′-methyl-CJ-17,572 (**7**).

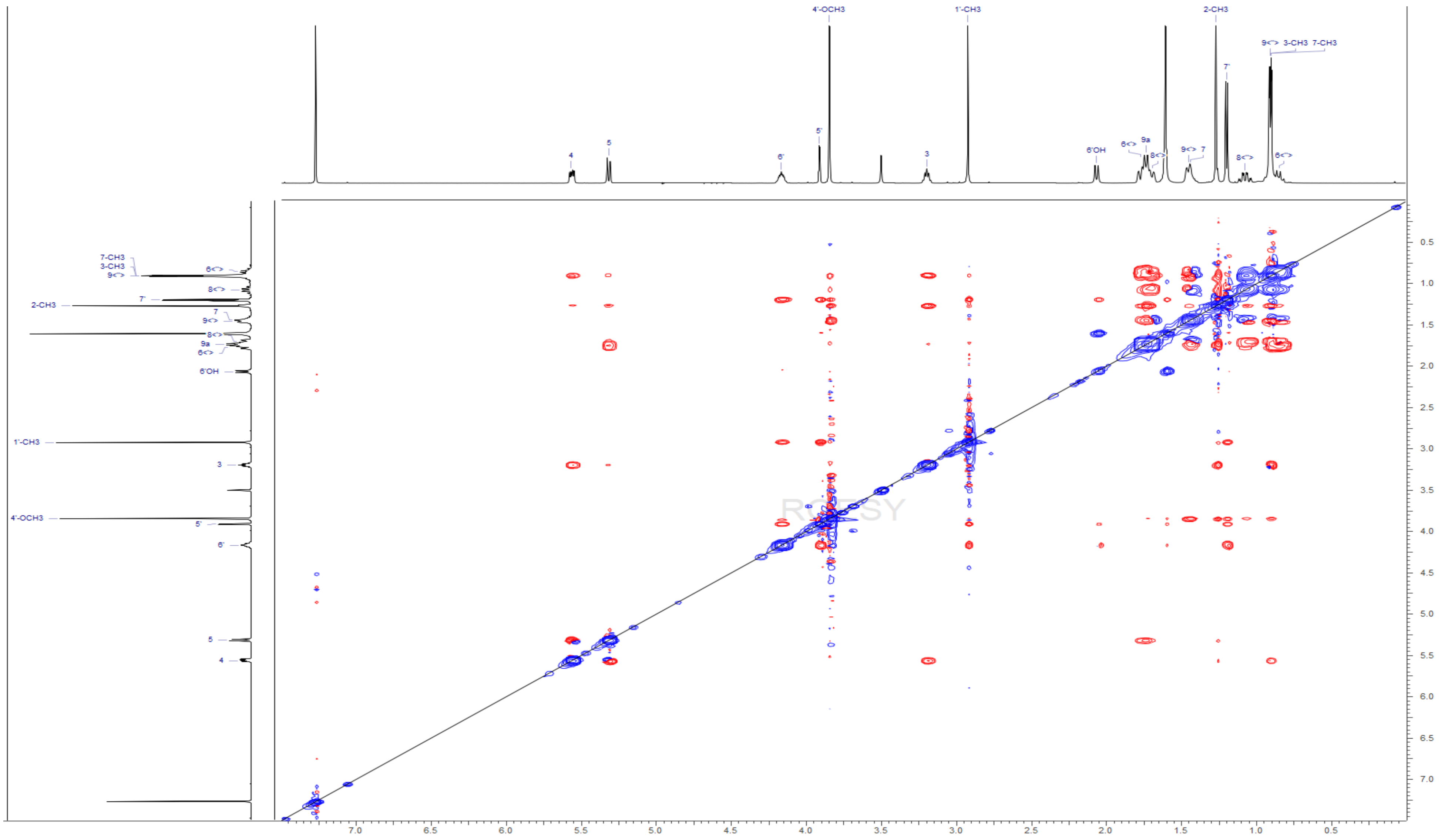

Figure S28: ROESY NMR spectrum (500 MHz, $CHCl_3$-*d*) of 4′-methyl-CJ-17,572 (**7**).

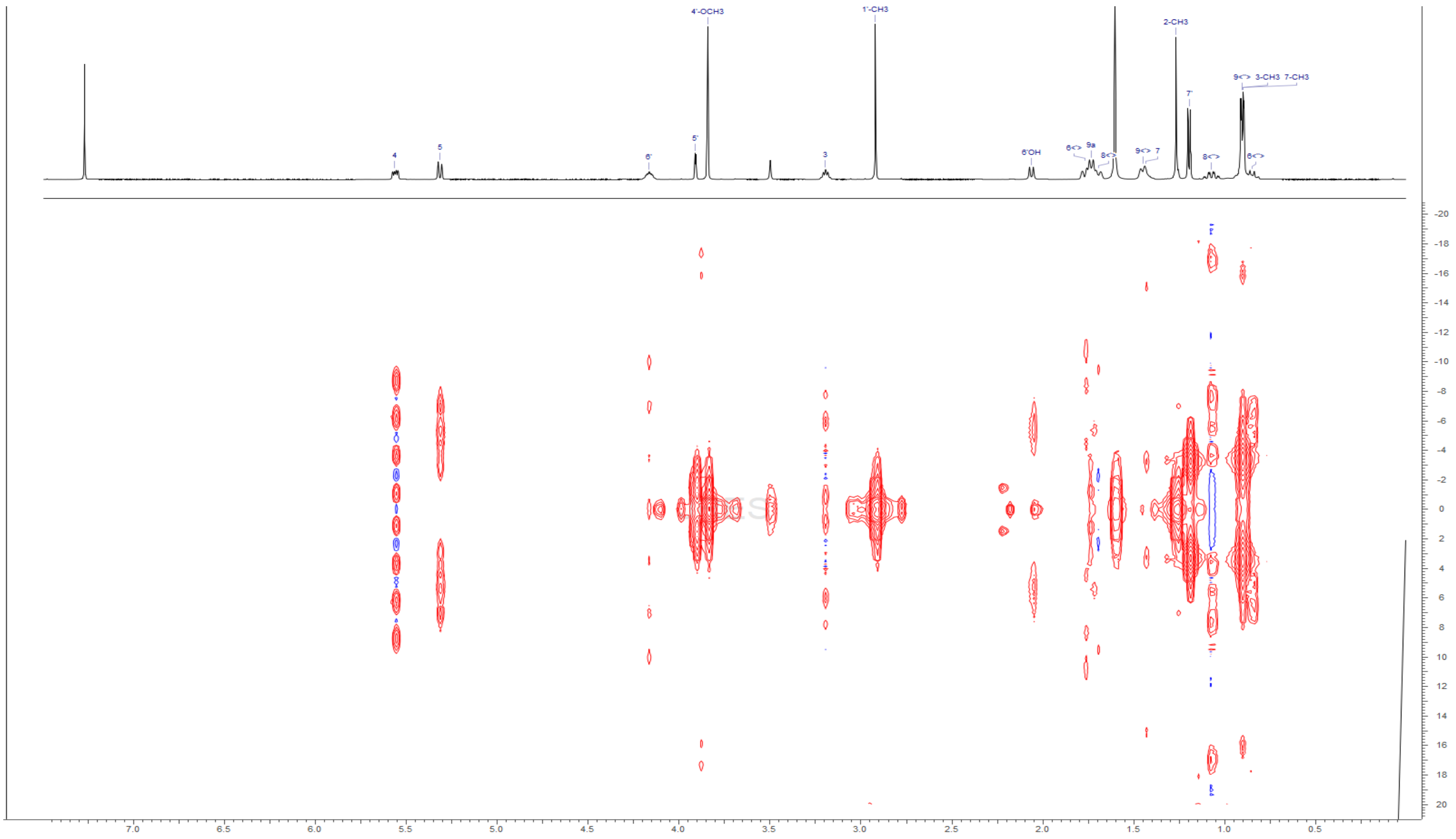

Figure S29: *J*-resolved NMR spectrum (700 MHz, CHCl3-*d*) of 4′-methyl-CJ-17,572 (**7**).

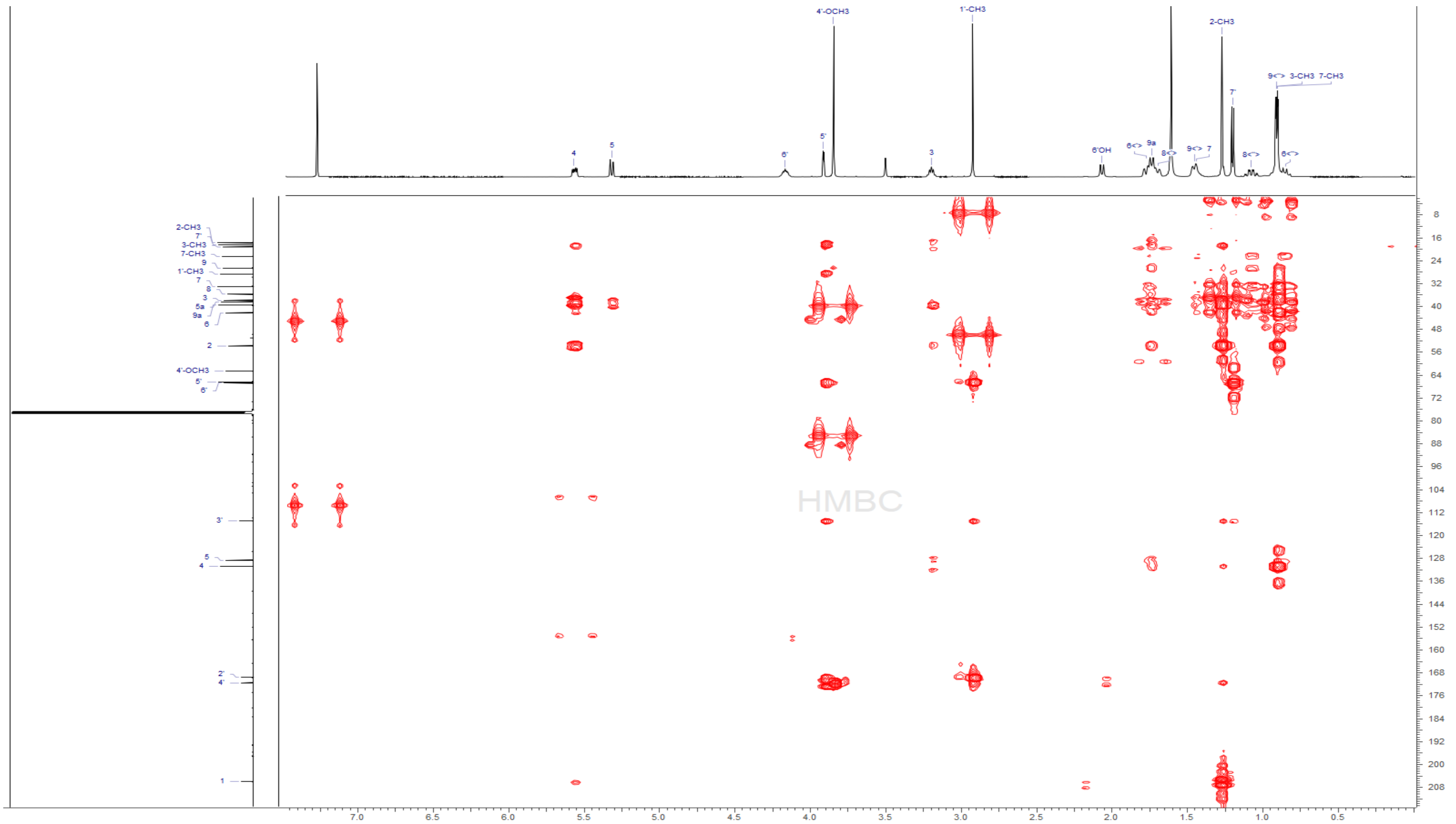

Figure S30: *J*-HMBC NMR spectrum (700 MHz, $CHCl_3$-*d*) of 4′-methyl-CJ-17,572 (**7**).

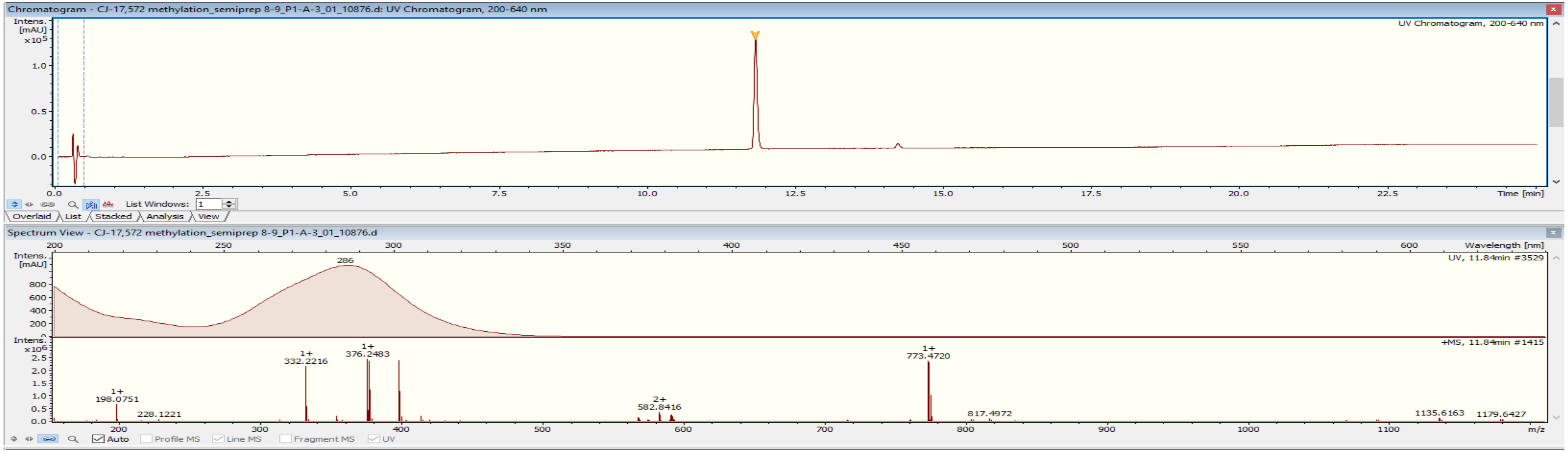

Figure S31: HR- ESI (+) MS data for 2′-methyl-CJ-17,572 (**8**).

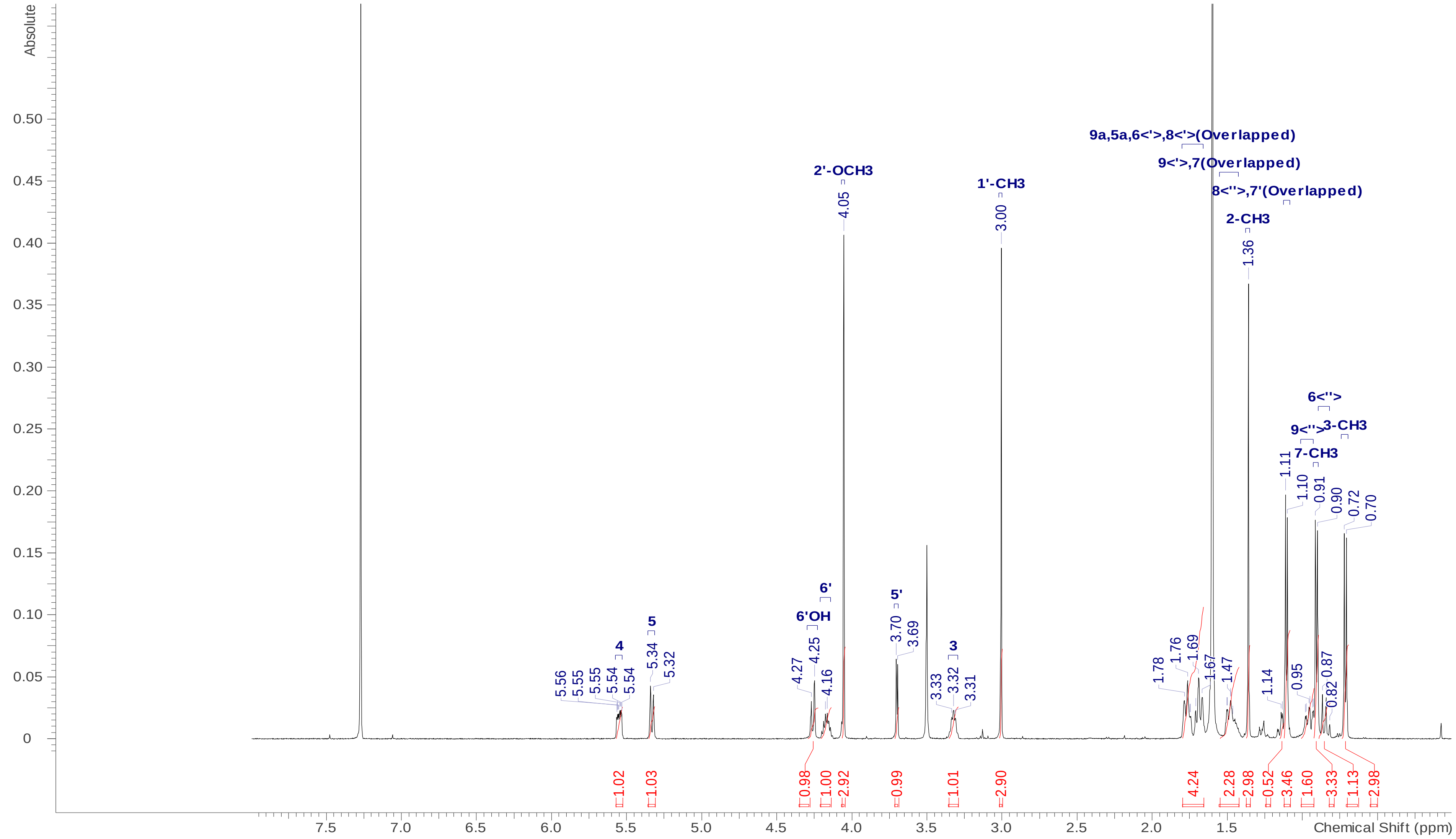

Figure S32: ^{1}H NMR spectrum (500 MHz, $CHCl_3$-*d*) of 2′-methyl-CJ-17,572 (**8**).

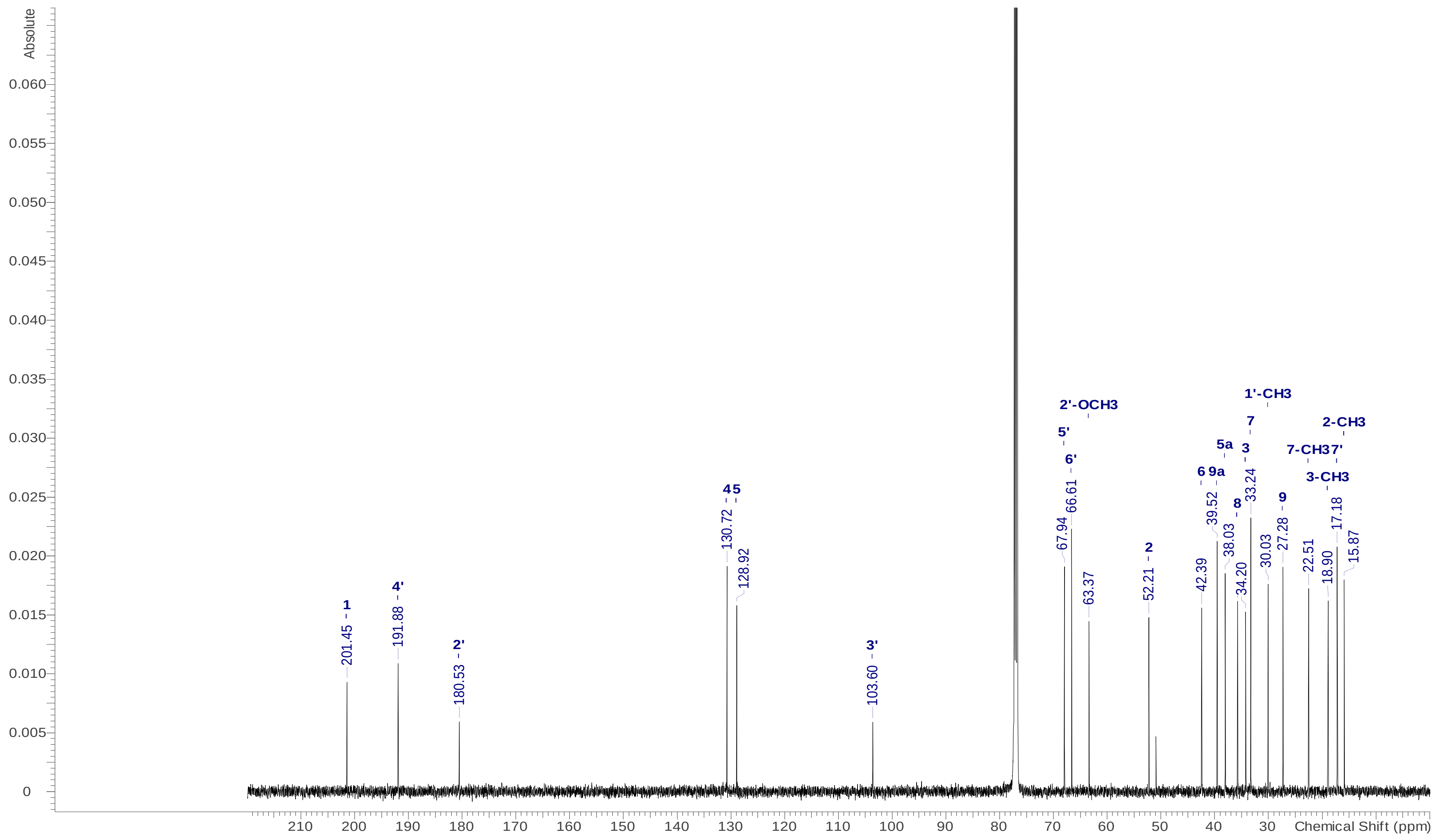

Figure S33: ^{13}C NMR spectrum (125 MHz, $CHCl_3$-*d*) of 2′-methyl-CJ-17,572 (**8**).

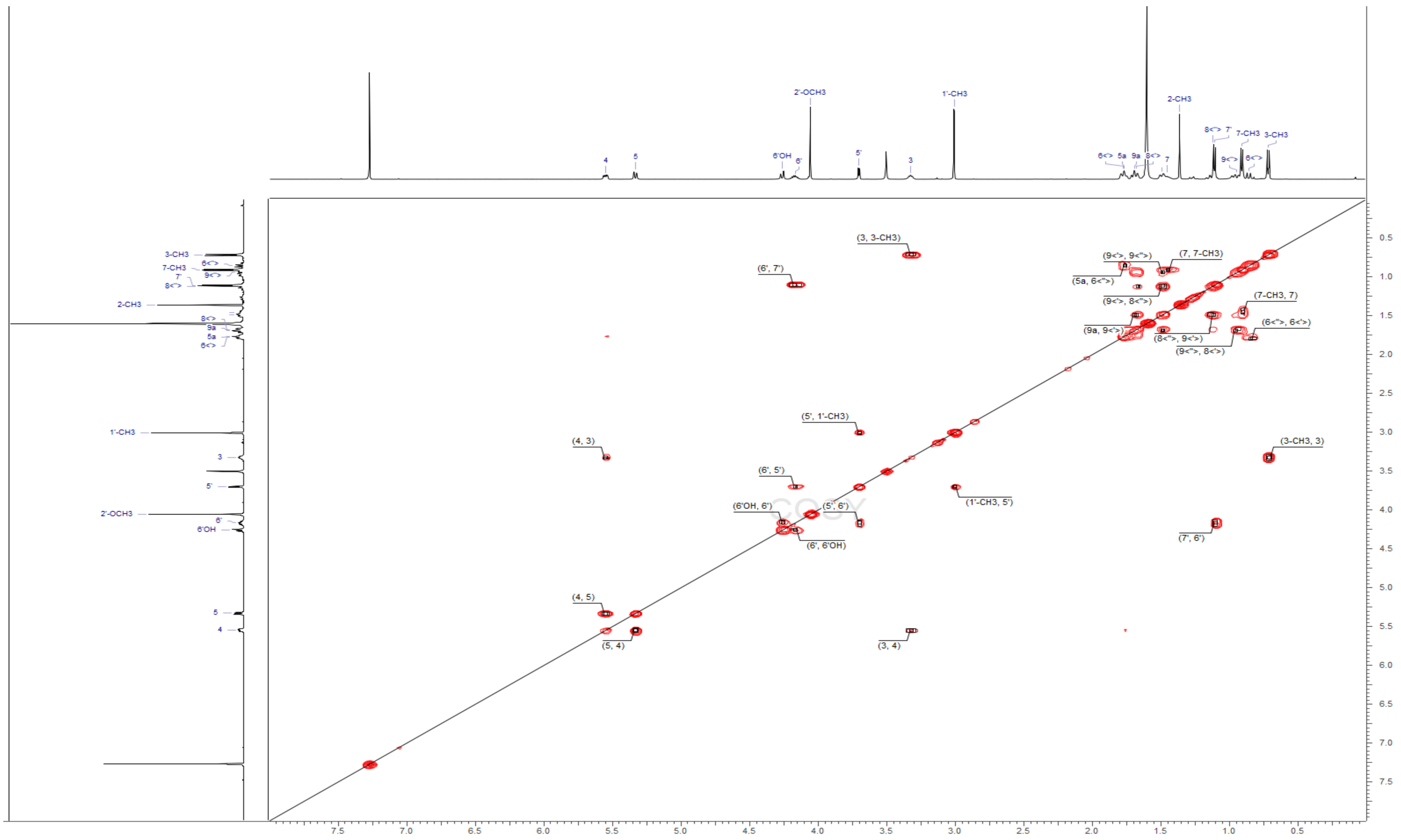

Figure S34: COSY NMR spectrum (500 MHz, $CHCl_3$-*d*) of 2′-methyl-CJ-17,572 (**8**).

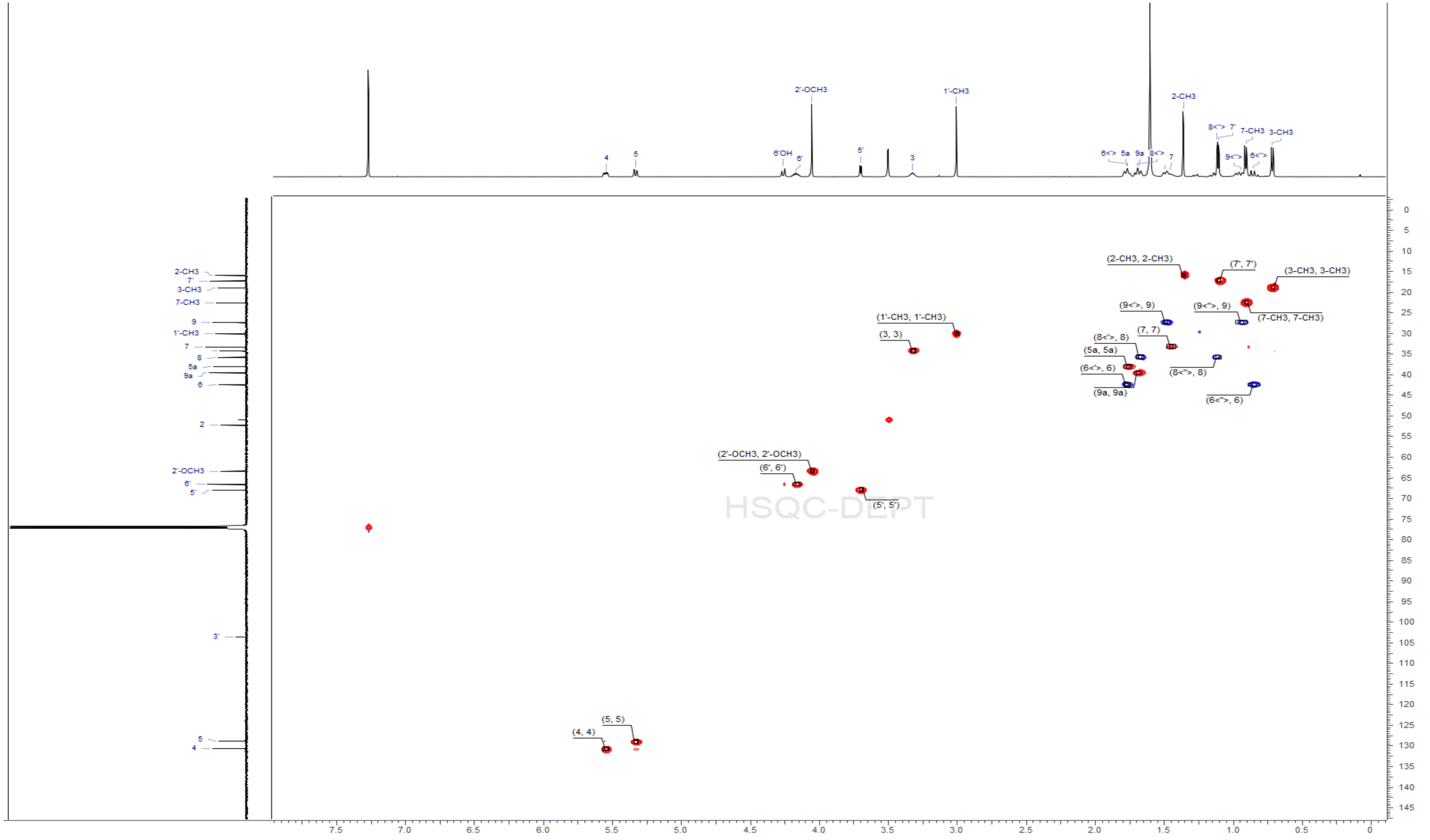

Figure S35: HSQC NMR spectrum (500 MHz, $CHCl_3$-*d*) of 2′-methyl-CJ-17,572 (**8**).

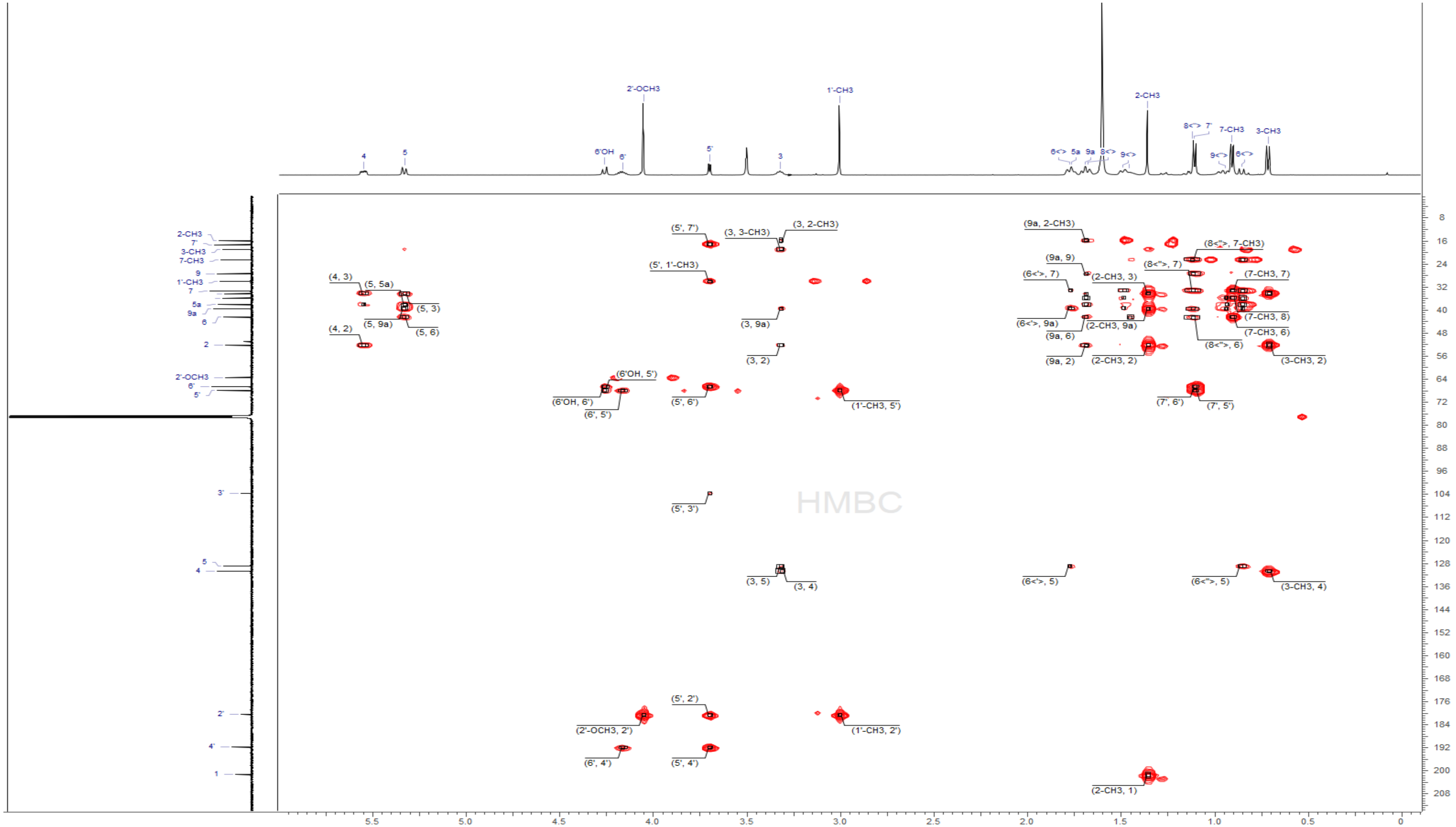

Figure S36: HMBC NMR spectrum (500 MHz, $CHCl_3$-*d*) of 2′-methyl-CJ-17,572 (**8**).

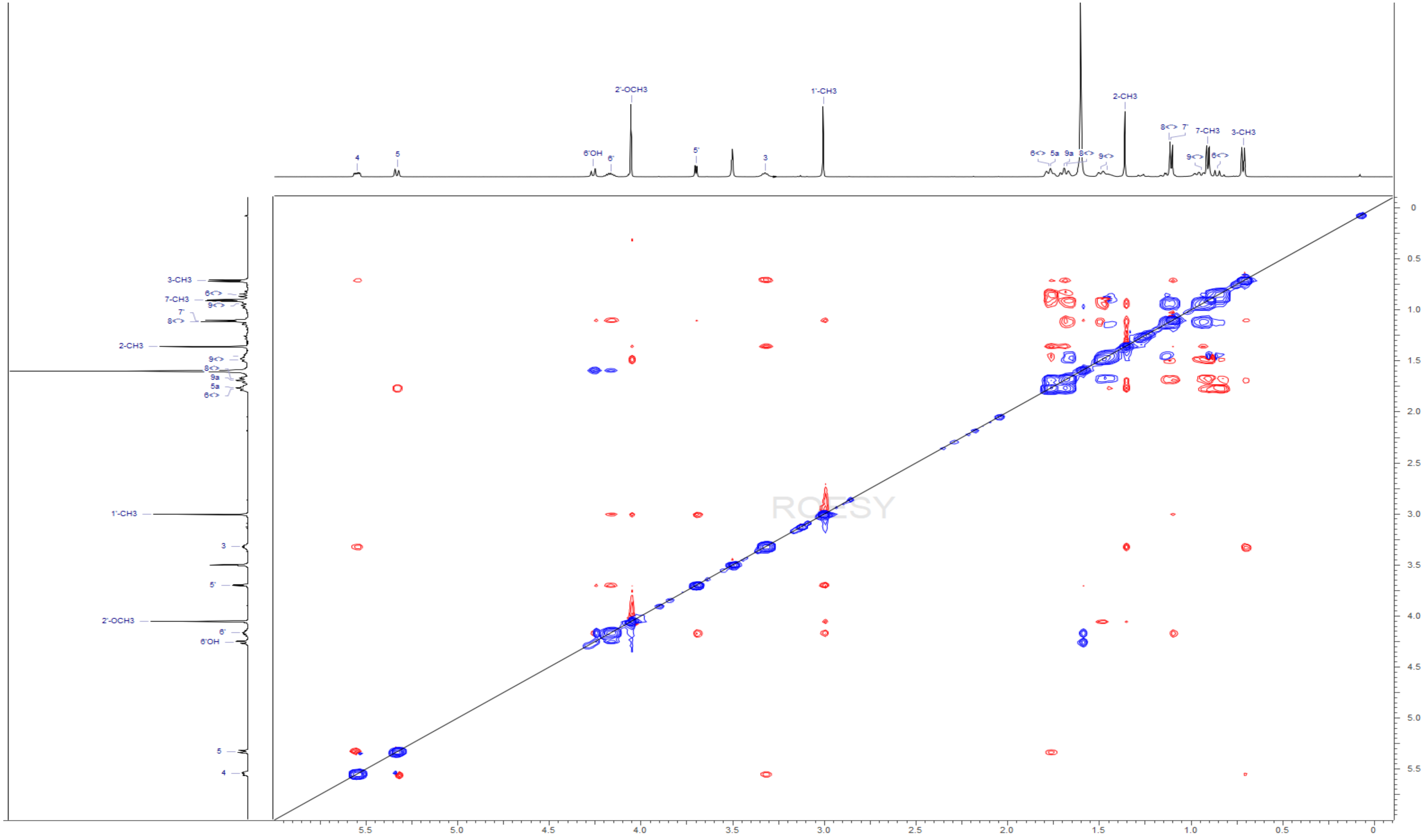

Figure S37: ROESY NMR spectrum (500 MHz, $CHCl_3$-*d*) of 2′-methyl-CJ-17,572 (**8**).

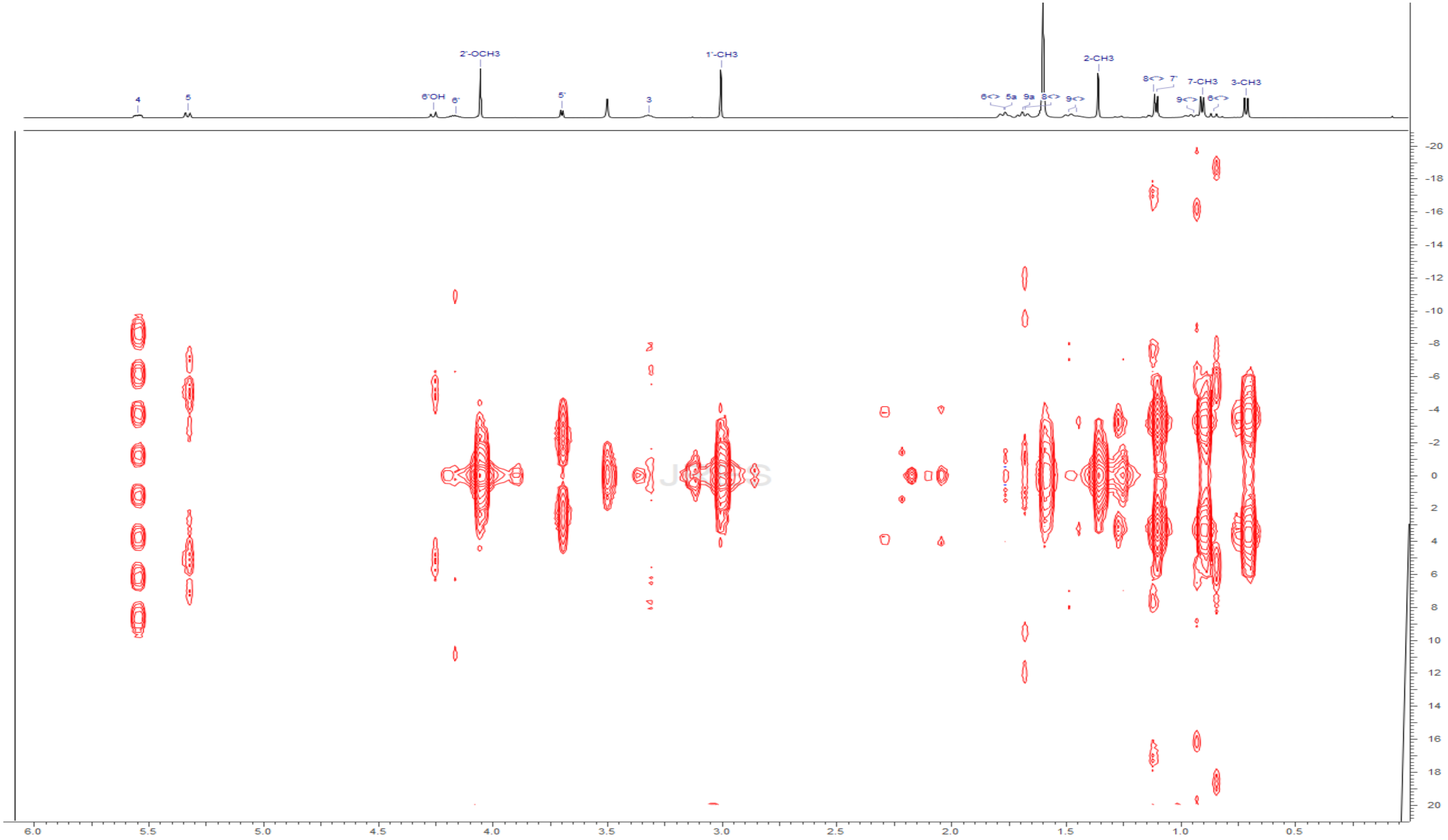

Figure S38: *J*-resolved NMR spectrum (500 MHz, $CHCl_3$-*d*) of 2′-methyl-CJ-17,572 (**8**)

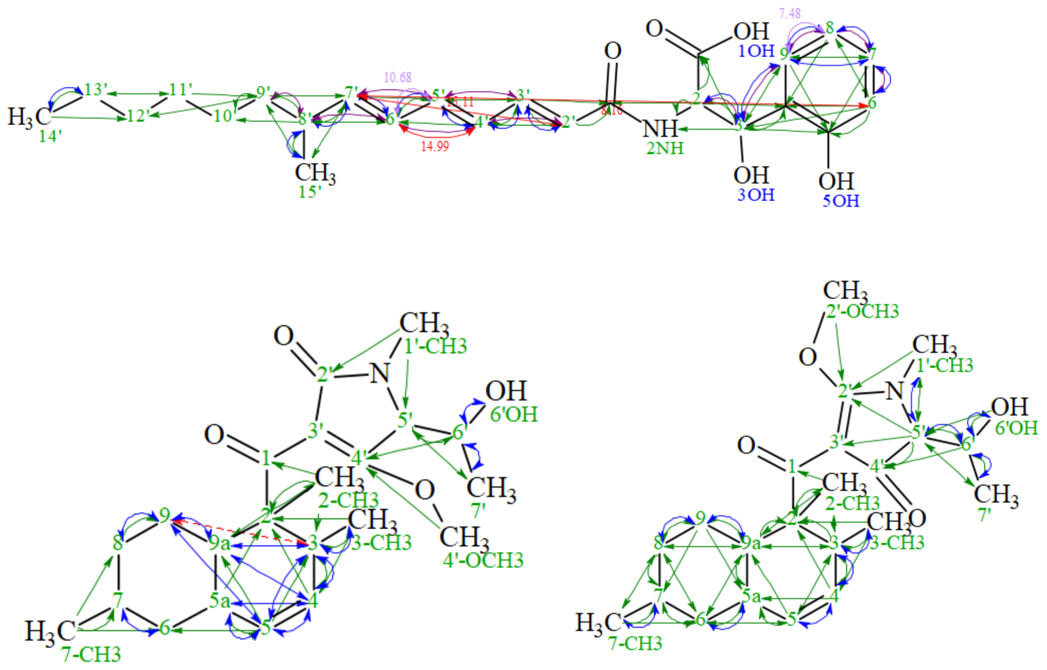

Figure S39: Indicative COSY/TOCSY (blue arrows), HMBC (green arrows) and ROESY correlations (violet arrows) for **2**, **7** and **8**.

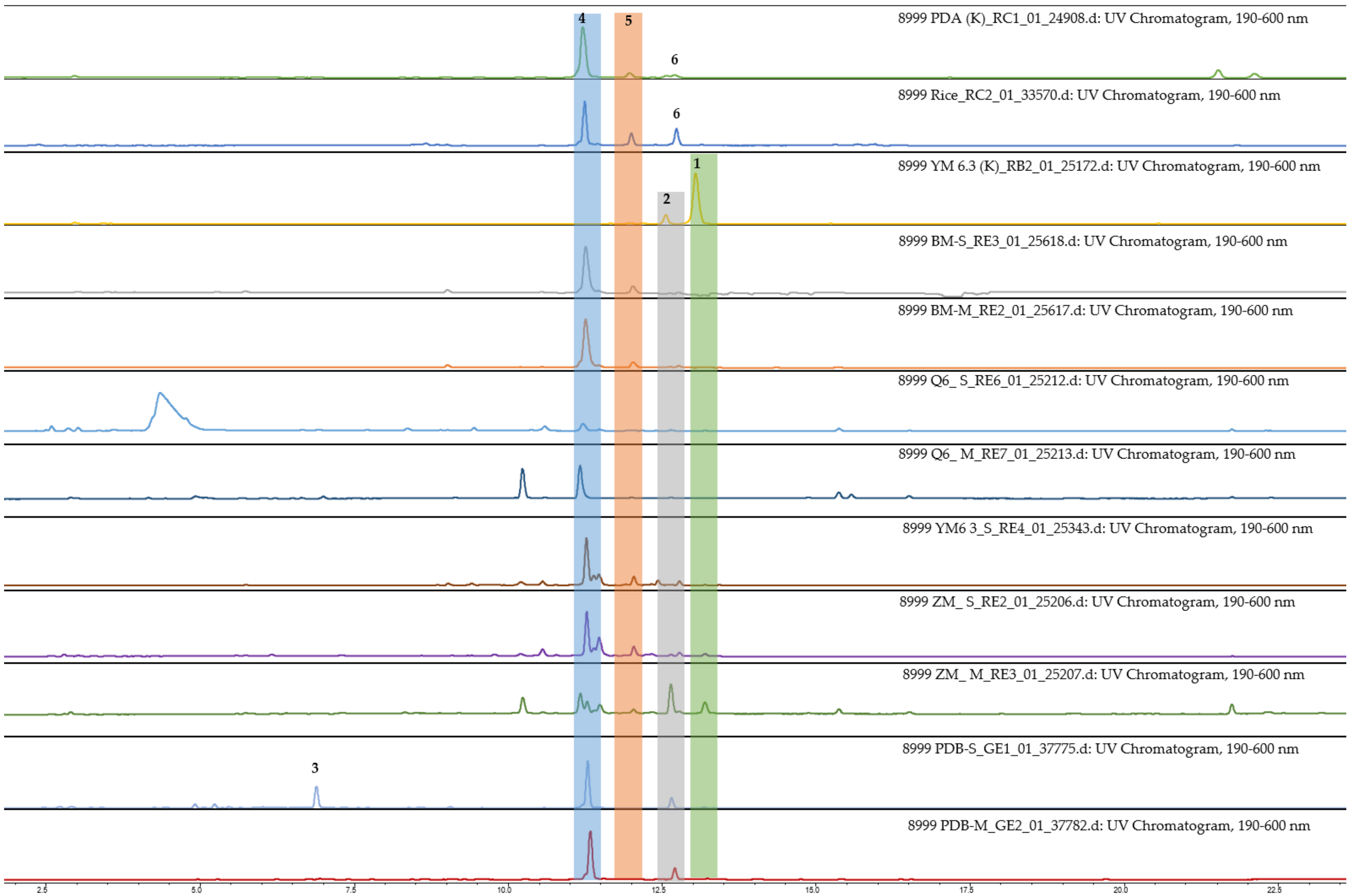

Figure S40: HPLC–UV/Vis chromatograms (at 190-600 nm) of the extracts from solid cultures (first 3 chromatograms) and extracts from liquid cultures (last 9 chromatograms).

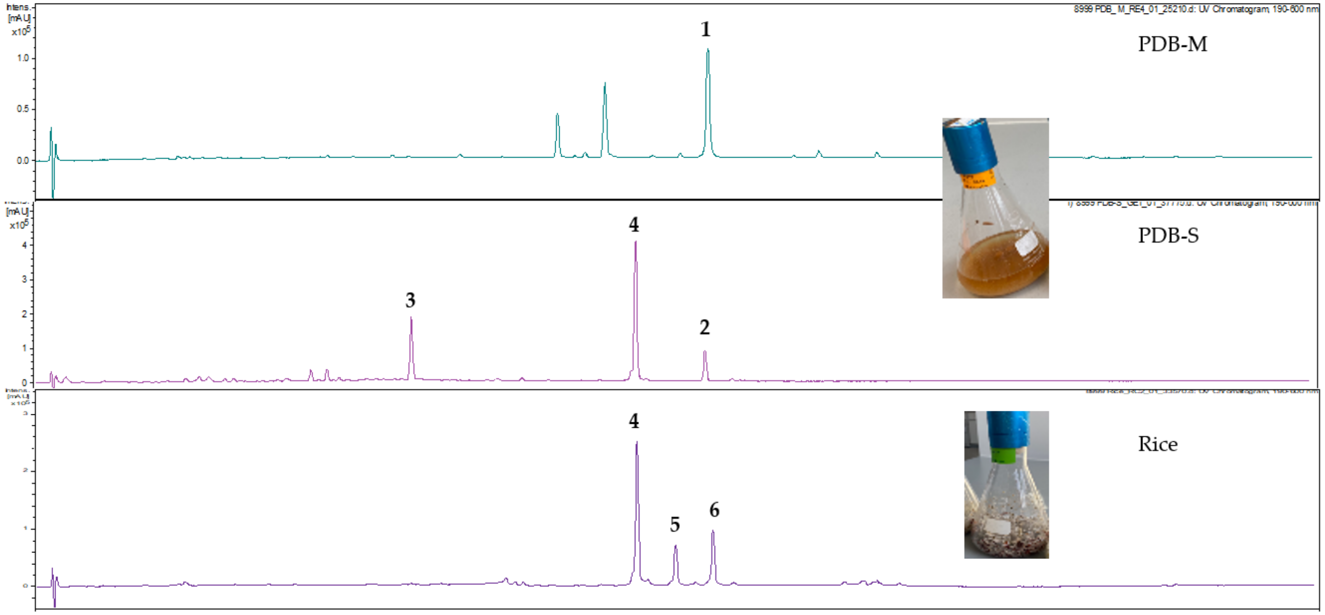

Figure S41: HPLC- UV/Vis chromatograms of the crude extracts of *Pezicula* sp. on/in selected media (at 190-600 nm).

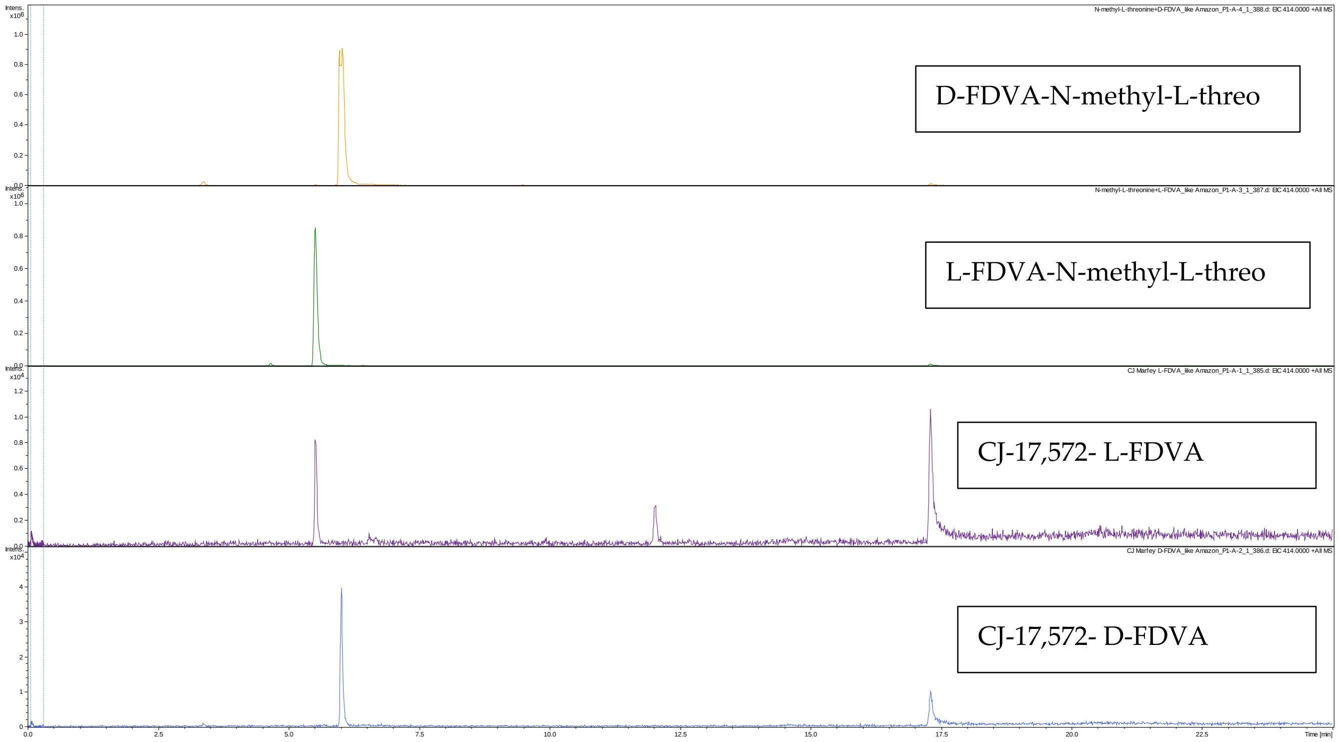

Figure S42: Extracted ion chromatograms (positive mode, *m*/*z*: 414.2) for Marfey's analysis.

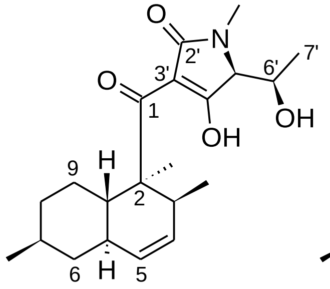

CJ-17,572 (1)

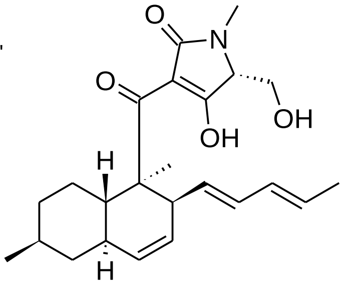

phomasetin

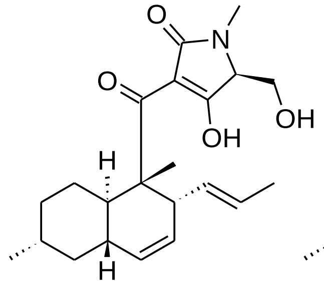

equisetin

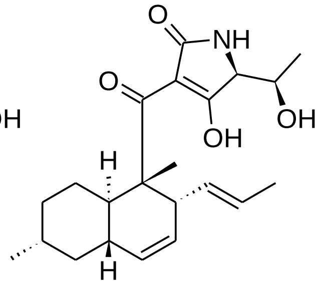

hymenosetin

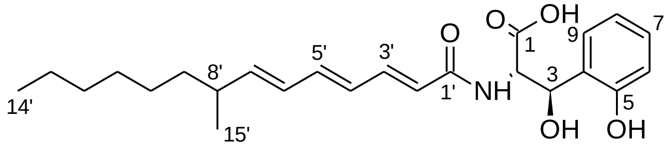

peziculastatin (2)

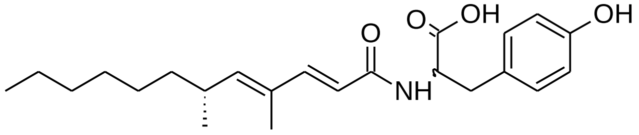

gymnastatin N

Figure S43: Comparison of CJ-17,572 (**1**) and peziculastatin (**2**) to structurally related compounds phomasetin, equisetin, hymenosetin and gymnastatin N.

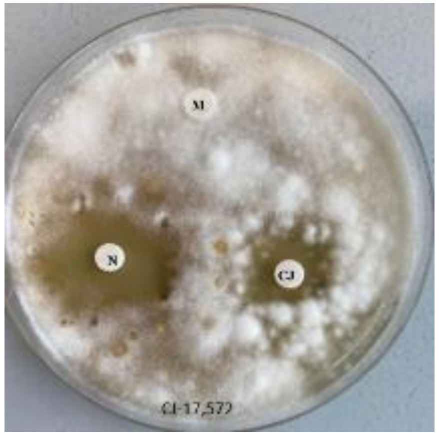

Figure S44: Agar disc (6 mm diam.) diffusion assay of compound CJ-17,572 (**1**) against *H. fraxineus* RH02-T6-B4-2, grown in 9-cm petri dishes on agar-based PD medium with *H. fraxineus*, N - positive control nystatin, M - negative control methanol, CJ – **1**.

Table S1. Antifungal activity of the compounds **1-6**. n.i.: no inhibition, n.t.: not tested.

**Compounds**	**Amount (µg/paper disc)**		
	**20**	**50**	**100**
CJ-17,572	9 mm	11 mm	n.t.
Peziculastatin	n.t.	n.i.	8 mm
Mycorrhizin A	19 mm	31 mm	n.t.
Cryptosporioptide A	n.t.	n.i.	n.i.
Cryptosporioptide B	n.t.	n.i.	8 mm
Cryptosporioptide C	n.t.	n.i.	n.i.
Nystatin	21 mm	24 mm	n.t.

Biofilm assays

To address inhibitory effects on biofilm of *P. aeruginosa* (PA 14), *P. aeruginosa* was precultured in 25 mL LB medium (Luria-Bertani Broth) filled in a 250 mL flask, incubated at 37 C and kept shaking at 100 rpm overnight. The OD_{600} of the suspended solution was measured and adjusted to 0.1 McFarland standard in M63 medium (supplemented with magnesium sulfate, glucose and casamino acids; see O'Toole 2011). The compounds were added into 150 µL bacterial solution at different concentrations (250–2 µg/mL). The solution was then added into U-bottom 96 well plates (Falcon non-tissue plate with U-Bottom ref.no 351177). The plates were incubated at 37 C at 150 rpm for 24 h to allow biofilm establishment at the air liquid interface. The plates were rinsed once with PBS and the biofilms stained with 150 µL 0.1% CV at room temperature for 15 min. After rinsing twice with PBS, the absorbance was quantified with a plate reader (Synergy 2, BioTek, Santa Clara, USA) at 550 nm using ethanol (95%). Methanol (2.5 %) and myxovalargin A (250–2 µg/mL) were used as negative and positive controls, respectively.

Table S2. Antibiofilm activity of compounds **4**–**6**.

**Compounds**	**Strains**	**Concentrations (µg/mL)**							
		**250**	**125**	**62.5**	**31.3**	**15.6**	**7.8**	**3.9**	**2**
**4**	***P. aeruginosa***	/	/	/	/	/	/	/	/
	**preformed *S. aureus***	80	73	70	/	/	/	/	/
	C. albicans	81	71	71	39	/	/	/	/
**5**	***P. aeruginosa***	/	/	/	/	/	/	/	/
	**preformed *S. aureus***	49	81	83	82	75	54	/	/
	C. albicans	74	50	49	/	/	/	/	/
**6**	***P. aeruginosa***	/	/	/	/	/	/	/	/
	**preformed *S. aureus***	23	77	78	78	72	68	49	39
	C. albicans	78	62	40	/	/	/	/	/
**MAA**	**preformed *S. aureus***	82	90	90	76	46	/	/	/
**Myxovalargin A**	***P. aeruginosa***	82	90	90	76	46	/	/	/
**Farnesol**	***C. albicans***	81	76	71	66	55	/	/	/

(/) no activity

Phytotoxicity assay

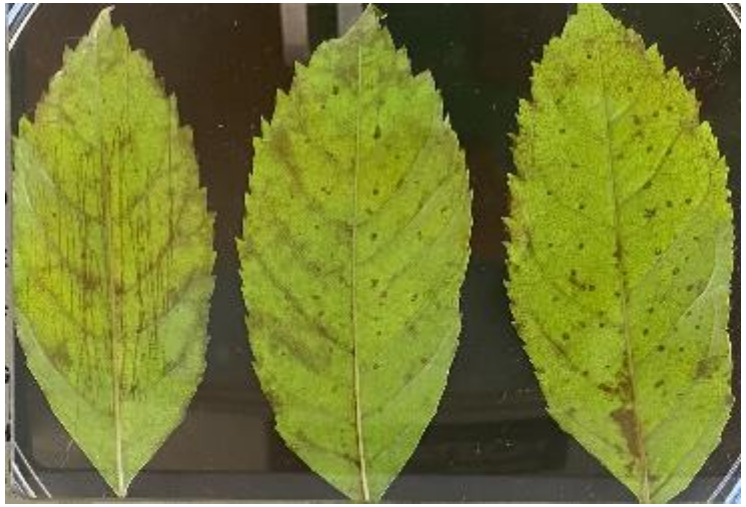

CJ-17,572

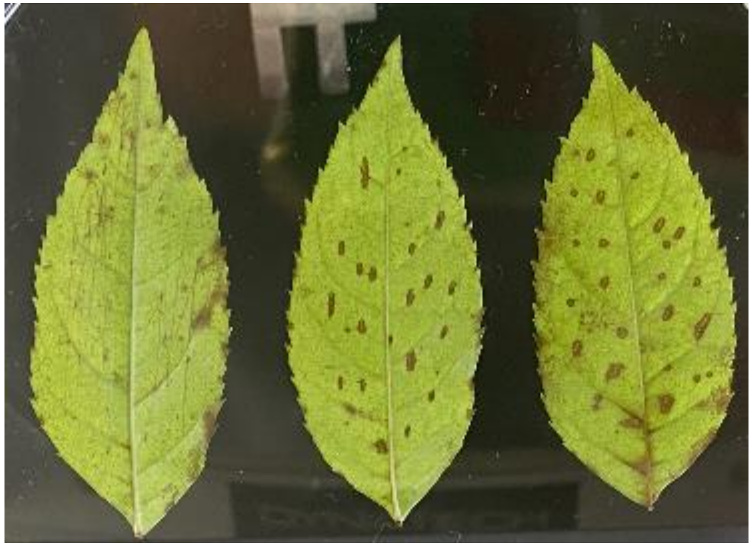

CJ-17,572

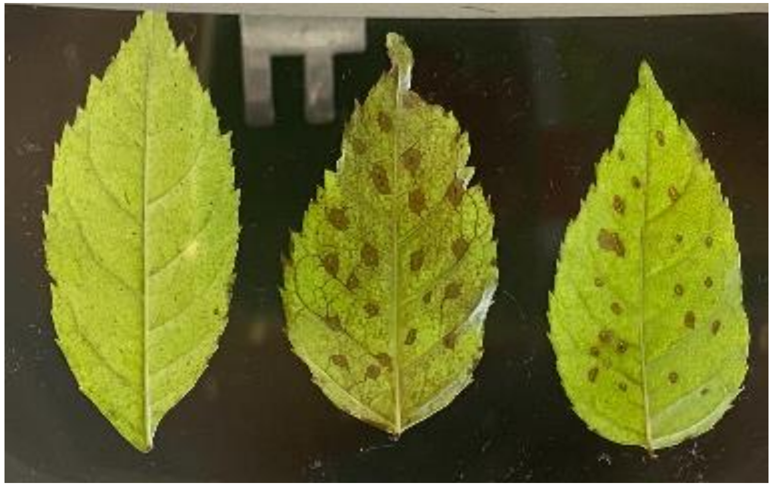

Mycorrhizin A

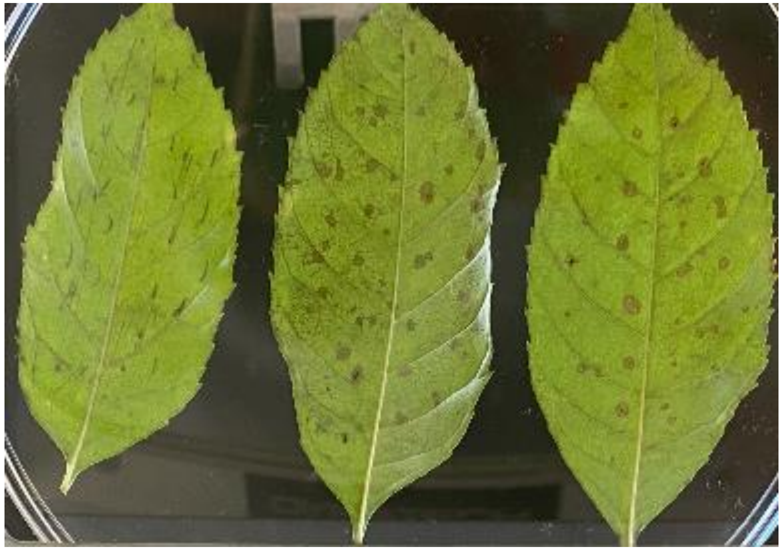

Mycorrhizin A

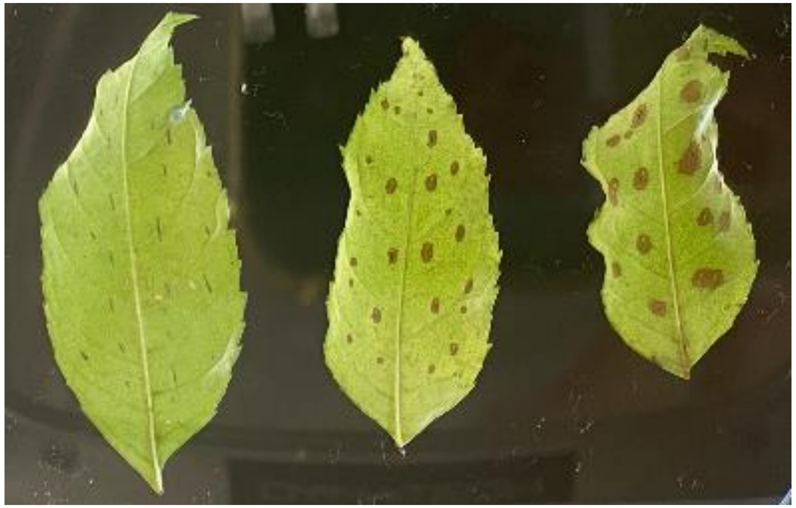

Cryptosporioptide A

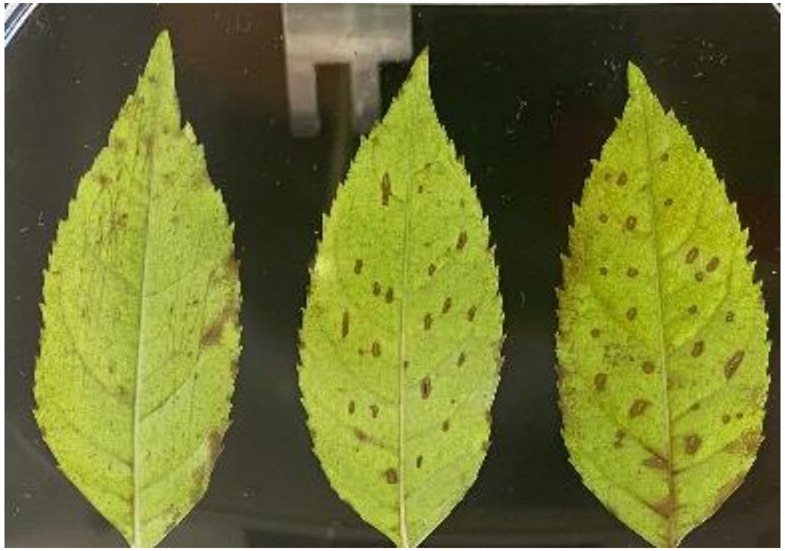

Cryptosporioptide A

Figure S45: Leaf puncture assay using CJ-17,572 (**1**), and mycorrhizin A (**3**) cryptosporioptide A (**4**). Each substance was tested on two leaves. Pictures show negative control 4% methanol (left), test substance (middle) and positive control macrocidin A (right).

Alignment of the ITS sequences used in this study:

>Pezicula_sp._DSM_110620

GCCAGCATCCTAGCCGAAGCGCGGTCCTCGGTCCCAGTAGGCTGCATTGCACCGAGGGCTATAACACTCCCCGAGAGGAGCCACATTCCCAAGGCCTTT
ATCCAACCACCGGAACCGATGCTGGCCTGAGCGAGGCCGAGTGCACCGGGGAGAACCCCGGATGATCAACCCCGCCCAAGTCTGGTTGCAAGCGCTTC
CCTTTCAACAATTTCACGTACTGTTTAACTCTCTTTCCAAAGTGCTTTTCATCTTTCGATCACTCTACTTGTGCGCTATCGGTCTCTGGCCAATATTTAGCTT
TAGATGAAATTTACCACCCATTTAGAGCTGCATTCCCAAACAACCCGACTCGTCGAAAGAGCTTTACACGGGCGCAGGCCACCGATCACATACGGGATT
CTCACCCTCTATGACGTCCTGTTCCAAGGAACTTAGACCGGGGCCGCACCCAAAGCATCTTCTACAAATTACAACTCGGAGCCTGAAAGACCCAGATTT
CAAATTTGAGCTGTTACCGCTTCACTCGCCGTTACTGAGGTAATCCCTGTTGGTTTCTTTTCCTCCGCTTATTGATATGCTTAAGTTCAGCGGGTATCCCTA
CCTGATCCGAGGTCAACCTTGATAAGTTGGGGGTTGCTGGCCAGCATCCACCGGGCGTCTGWAACGAGAGTCTGTACTACGCTTAGGCATCGATGAAG
AACG
